# Restriction on self-renewing asymmetric division is coupled to terminal asymmetric division in the Drosophila CNS

**DOI:** 10.1371/journal.pgen.1009011

**Published:** 2020-09-28

**Authors:** Ivana Gaziova, Michael Gazi, Jordan Mar, Krishna Moorthi Bhat

**Affiliations:** 1 Department of Neuroscience and Cell Biology, University of Texas Medical Branch, Galveston, United States of America; 2 Texas Biomedical Research Institute, Department of Virology, 8715 W. Military Dr. San Antonio, United States of America; 3 Department of Molecular Medicine, Morsani College of Medicine, University of South Florida, Tampa, United States of America; New York University, UNITED STATES

## Abstract

Neuronal precursor cells undergo self-renewing and non-self-renewing asymmetric divisions to generate a large number of neurons of distinct identities. In Drosophila, primary precursor neuroblasts undergo a varying number of self-renewing asymmetric divisions, with one known exception, the MP2 lineage, which undergoes just one terminal asymmetric division similar to the secondary precursor cells. The mechanism and the genes that regulate the transition from self-renewing to non-self-renewing asymmetric division or the number of times a precursor divides is unknown. Here, we show that the T-box transcription factor, Midline (Mid), couples these events. We find that in *mid* loss of function mutants, MP2 undergoes additional self-renewing asymmetric divisions, the identity of progeny neurons generated dependent upon Numb localization in the parent MP2. MP2 expresses Mid transiently and an over-expression of *mid* in MP2 can block its division. The mechanism which directs the self-renewing asymmetric division of MP2 in *mid* involves an upregulation of Cyclin E. Our results indicate that Mid inhibits *cyclin E* gene expression by binding to a variant Mid-binding site in the *cyclin E* promoter and represses its expression without entirely abolishing it. Consistent with this, over-expression of *cyclin E* in MP2 causes its multiple self-renewing asymmetric division. These results reveal a Mid-regulated pathway that restricts the self-renewing asymmetric division potential of cells via inhibiting *cyclin E* and facilitating their exit from cell cycle.

## Author summary

Nerve cells in the brain, spinal cord, gut and so on in all organisms are generated from stem cells. These primary cells divide to self-renew and at the same time generate a secondary precursor cell that terminally divides to produce two cells that differentiate into neurons of different identities, or glial cells or a neuron and a glia. The secondary cells never self-renew, the reason for which is not known. We found that in embryos that lack the activity of a gene called *midline*, precursors such as MP2 that normally divides into two neurons, self-renews and generates a neuron at the same time. The identity of the differentiating progeny is tied to how the asymmetrically localized determinant Numb is distributed in the precursor cell. When this gene, *midline*, is over expressed, it blocks MP2 division. The way Midline protein works is that it represses the *cyclin E* gene via binding to sites in its promoter, preventing the over-expression of Cyclin E and thus blocking cells from entering the cell cycle. A deregulation of *cyclin E* as in loss of function *midline* mutants allows one of the daughter cells of MP2 to re-enter cell cycle as MP2, just as an over-expression of the *cyclin E* gene also does. These results show a mechanism by which restriction on self-renewing asymmetric division is coupled to terminal asymmetric division and works through Midline and Cyclin E. This work addresses one of the fundamental problems is biology.

## Introduction

The broad problem of how the division potential of cells is controlled during development is highly significant. The regulation of the division potential of neural precursors, and their asymmetric division, with or without self-renewal, are fundamental processes that govern the formation of a functional CNS in all animals. The Drosophila model system offers one of the best systems to explore this issue given the availability of mutations and genetic tools [1–5]. While we have made much progress in understanding the biology of stemness and asymmetric division of precursor cells [reviewed in ref. 6], almost nothing is known about the regulation of division potential, a process of great importance. Too few or too many divisions of precursor cells will leave the CNS aberrant and dysfunctional. We sought to use the development of the CNS in Drosophila as a paradigm to study both the regulation of division potential and how this is tied to precursor cell asymmetric division.

During neurogenesis in Drosophila, a large number of neurons are generated within the CNS via two types of precursor cells, each type undergoes a distinct kind of asymmetric division [1, 2]. The first type of precursor cell is the primary neuronal precursor or neuroblast (NB). NBs typically undergo a varying number of self-renewing asymmetric divisions, a fundamental property of all stem cells. The second type of precursor is the secondary neuronal precursor or ganglion mother cell (GMC). These cells undergo a single terminal asymmetric division without any self-renewal. This division generates two distinct post-mitotic neurons. Thus, these two types of divisions generate a large number of neurons of different identities from a few founder cells.

NB stem cells in the CNS divide a varying number of times during development, from one to as many as 18 (or perhaps even more), before becoming quiescent or post-mitotic or possibly die [2, 3]; some of the quiescent NBs re-enter the cell cycle during larval or pupal stages. At least one NB, known as MP2, while formed as a NB during the first of the five waves of NB delamination under the control of neurogenic and proneural genes similar to other NBs, it behaves as a GMC and divides only once to produce a pair of post-mitotic neurons [2, 7]. Some NBs are believed to behave similar to MP2 in their final round of division, thus, other NBs become quiescent to re-enter cell cycle in larval or pupal stages, and some may even die.

We have a good understanding of how a terminal asymmetric division of MP2 or a GMC generates two different neurons. This is achieved through asymmetric localization of determinants such as Inscuteable (Insc), Notch, Numb, Prospero, Neuralized, etc [7–17]. However, we do not know how a self-renewing asymmetric division is regulated. It appears that this is tied to the specification of the division potential of a specific NB. It is not known what genes restrict or specify NB division potential. Similarly, the mechanism and genes that restrict MP2 division to just one round are not known/understood. This is a significant problem in neurobiology as this timing and restriction or limit on the division potential of precursors form the basis for producing specific and correct number of neurons from flies to humans to form a functional CNS. We think that loss of function for genes involved in these processes will result in precursors such as MP2 and other NBs and GMCs undergoing additional rounds of division.

During the course of our work, we found that loss of function for *midline*, also known as *lost in space* (*los*) or *extra* [18–21] causes additional divisions of precursor cells, and therefore a candidate for regulating the division potential of precursors. Mid is a transcription factor belonging to the class of proteins known as T-box binding (Tbx) proteins. These proteins are highly conserved and have a 180–230 amino acid DNA-binding T-box domain. They bind to a 20-bp degenerate palindromic sequence called T-Box element (TBE) [22]. TBEs are highly variable in sequence, number and distribution within promoters and Tbx proteins diverge in their sequence preference as well [20]. Tbx proteins appear to repress transcription of genes [20, 23]. In vertebrates, haploinsufficiency for mouse brachyury (Tbx protein) and human TBX3 and TBX1 genes causes dominant phenotypes such as short tails/tailless, Ulnar-Mammary syndrome and DiGeorge syndrome, respectively [24, 25]. Upper limb malformation and congenital heart defects in Holt-Oram syndrome are due to haploinsufficiency for TBX5 [24, 26–28]. Thus, developmental processes appear to be sensitive to the levels of Tbx proteins.

In *Drosophila*, loss of function for *mid* causes cuticle defects in the midline region of the embryo, thus the name *midline* [18]. Loss of function for *mid* also causes defects in the lateral chordotonal axons, shorter and defasciculated dorsally routed axons in the peripheral nervous system (PNS)[19]. We have also shown that Mid is involved in identity specification of NBs and GMCs and regulate axon guidance via blocking the reiteration of the identity of rows of NBs within the ventral nerve cord [20, 21]. These results argue that Mid is involved in several important decision-making steps during CNS development.

In this work, we present evidence that Mid regulates the division potential of precursor cells such as MP2 and at least one GMC lineage. In MP2, Mid appears to couple restriction on self-renewing asymmetric division with terminal asymmetric division. This involves regulation of the *cyclin E* gene and localization of the Numb protein in precursor cells. Mid represses *cyclin E* by binding to the TBE within the *cyclin E* promoter. Cyclin E is the upstream regulator of the transition from G1 to S [29–33]. This is consistent with the finding that the temporal abundance of Mid in precursors changes in a dynamic manner, enabling it to carry certain temporal information. Our results allow us to build a model in MP2 where Mid peaks at a late stage MP2 and as a consequence, Cyclin E is downregulated. Thus, when MP2 divides into two post-mitotic neurons, Cyclin E level is kept below the threshold level required for entry to S-phase, and cells exit cell cycle. In the absence of Mid, *cyclin E* is de-repressed in MP2 and the high levels of Cyclin E pushes one or more progeny cells to enter S-phase, and thus, the cell cycle. Via an additional role for Mid in the localization of asymmetric determinants such as Numb, different progeny neurons are generated during lineage elaboration. While Mid represses *cyclin E*, this repression is not complete or an ON/OFF situation and this appears to have been achieved via two ways: one, by having variants of the Mid binding sites in the *cyclin E* promoter such that the repression is meant to prevent an over-expression of *cyclin E* but not abolish it, and two, by tightly regulating the levels of Mid itself, the mechanism for which is not yet known.

## Results

### Loss of function for Mid causes additional self-renewing asymmetric divisions in the MP2 lineage

**Fig 1:**
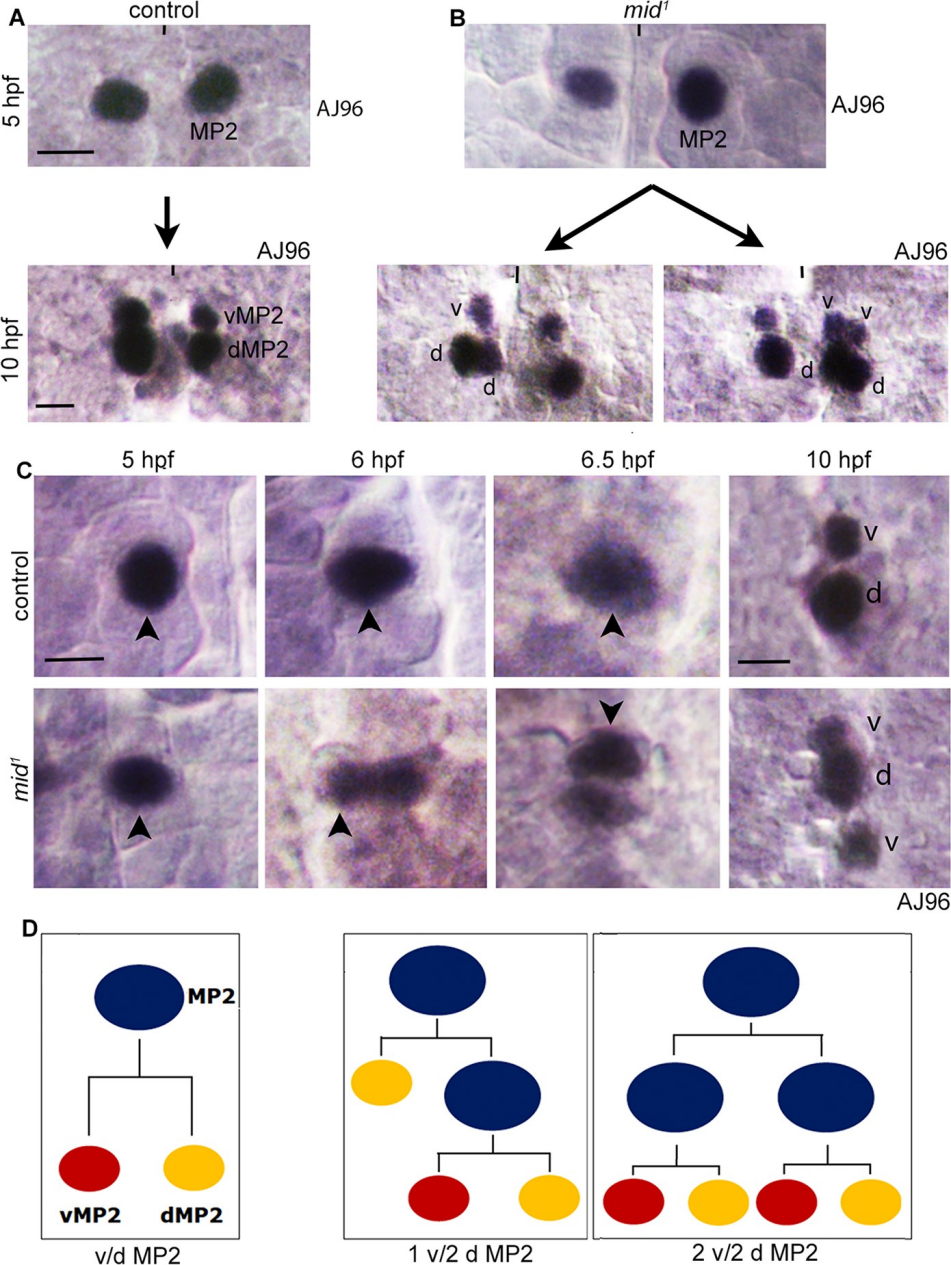
Extra divisions in the MP2 lineage in *mid* mutant embryos. The anterior end is up, the midline is marked by vertical lines. Only one segment is shown. See Supplementary Information for the dataset (see S1 Data). **(A and B):** Wild-type control and *mid* mutant embryos were stained for AJ96, which is an enhancer-trap line and is expressed in MP2, vMP2 (v) and dMP2 (d). In control (A), an MP2 generates a v and a dMP2. In *mid* (B) only one MP2 is formed but it generates extra v and d MP2 neurons: examples of a 2d/v and a 2d/2v MP2 neurons are shown. About 99% of the hemisegments had only one MP2 in control or the mutant (see Table 1). Magnification bar: 10 μm (top panels) and 5 μm (bottom panels), respectively. **(C):** Control and *mid* mutant embryos of different developmental ages were stained for AJ96 expression to determine the MP2 division pattern. Only one AJ96-expressing MP2 was observed in control or *mid* mutant embryos per hemisegment. MP2 forms around 4 hpf and divides around 6.5 hpf. MP2 in the mutant appears to divide around 6 hpf, therefore sooner than the division in control. By 6.5 hpf, the MP2 in *mid* has divided whereas in the control it appears ready to divide. By 10 hpf, only a v and a d MP2 were seen in the control where as two vMP2s and one dMP2 were seen in the mutant in this hemisegment. One half-segment is shown in each panel. At least 16 hemisegments were analyzed from 4 different embryos. Magnification bar: 10 μm in 5 hpf-6.5 hpf panels, and 5 μm in 10 hpf panels. **(D):** The line drawings of MP2 lineage elaborations in wild-type control and *mid* mutant embryos.

MP2 is a primary precursor formed as an S1 NB in row 3, medial column around 4 hours post-fertilization (hpf)[2]. An MP2, unlike other NBs, undergoes only one terminal asymmetric division ~6.5 hpf, generating a dorsally, posteriorly located larger dMP2, and a ventrally, anteriorly located smaller vMP2 (Fig 1A). In *mid* los of function mutant embryos (*mid*^*1*^ *mid*^*los1*^, *mid*^*extra*^ or *mid*, *H15*^*df*^, which is the same as *mid*^*df*^ and removes both *mid* and its sister gene *H15*), we found that the MP2 lineage was affected. This was first observed with AJ96, an enhancer-trap marker for the MP2 lineage and is expressed in MP2, v and dMP2 (Fig 1) [34]. While only one MP2 per hemisegment was observed in 5 hpf *mid* mutant embryos (Fig 1B) as in wild-type control (Fig 1A), additional d and vMP2 neurons per hemisegment were observed in 10 hpf *mid* mutant embryos (Fig 1B). These neurons were one vMP2 and 2 dMP2s, or 2 vMP2 and 2 dMP2 or 2 vMP2 and one dMP2 (see also Table 1). Extra v/d MP2s could be generated via a symmetrical division of MP2 (Fig 1D), or formation of a second MP2 from the equivalence group or transformation of another NB into MP2. These instances will generate a four cells-phenotype (even number of neurons). However, in *mid*, the most frequent phenotype was a 3-cells phenotype, the first one generated via a self-renewing asymmetric division of MP2, and the two additional ones by the terminal asymmetric division of the self-renewed MP2 (Fig 1D).

**Table 1:** Penetrance of the MP2 lineage defects in *mid* mutant embryos.

% of hemisegments with									
	Missing/dup. MP2	v/dMP2 pairs	2 dMP2/ 1 vMP2	1 dMP2/ 2 vMP2	2 dMP2/ 2 vMP2	2 dMP2	2 vMP2	>2 dMP2	>2 vMP2
control	2.0 ± 0.7	99 ± 0.6	0	0	0	0	0	0	0
*mid*^*df*^	1.7 ± 0.3	40 ± 1.7	26 ±2.2	5 ± 1.2	12 ± 1.9	9 ±1.1	2.7 ± 1.4	3.0±1.2	0

For analyzing the missing and duplication of MP2 lineage, we used Ac staining (this was also confirmed with AJ96-lacZ staining, which gave similar penetrance). For the analysis of v/d MP2 defects, AJ96 and Odd markers were used. Missing/duplication of MP2 were analyzed in embryos that were between 4.5–6.5 hpf, to examine the v/d progeny neurons, embryos from 6.5 hpf to 14 hpf were examined. See Supplementary Information (S1 Data).

We explored this issue in more detail by staining embryos fixed at different time-points to track the formation and division of an MP2 in a time-lapse manner. As shown in Fig 1C, we did not observe two MP2s but only one in the mutant as in wild-type control. But, the MP2 in the mutant divided slightly earlier than in control (~6 hpf versus 6.5 hpf). By 10 hpf there were 3 cells, 2 vMPs and one dMP2 in the mutant versus a d and a vMP2 in control (Fig 1C). These results suggest that the development of the MP2 lineage might be hastened in *mid* mutant embryos to generate additional progeny neurons via a self-renewing asymmetric division. The division patterns in *mid* embryos are summarized in Fig 1D.

**Fig 2:**
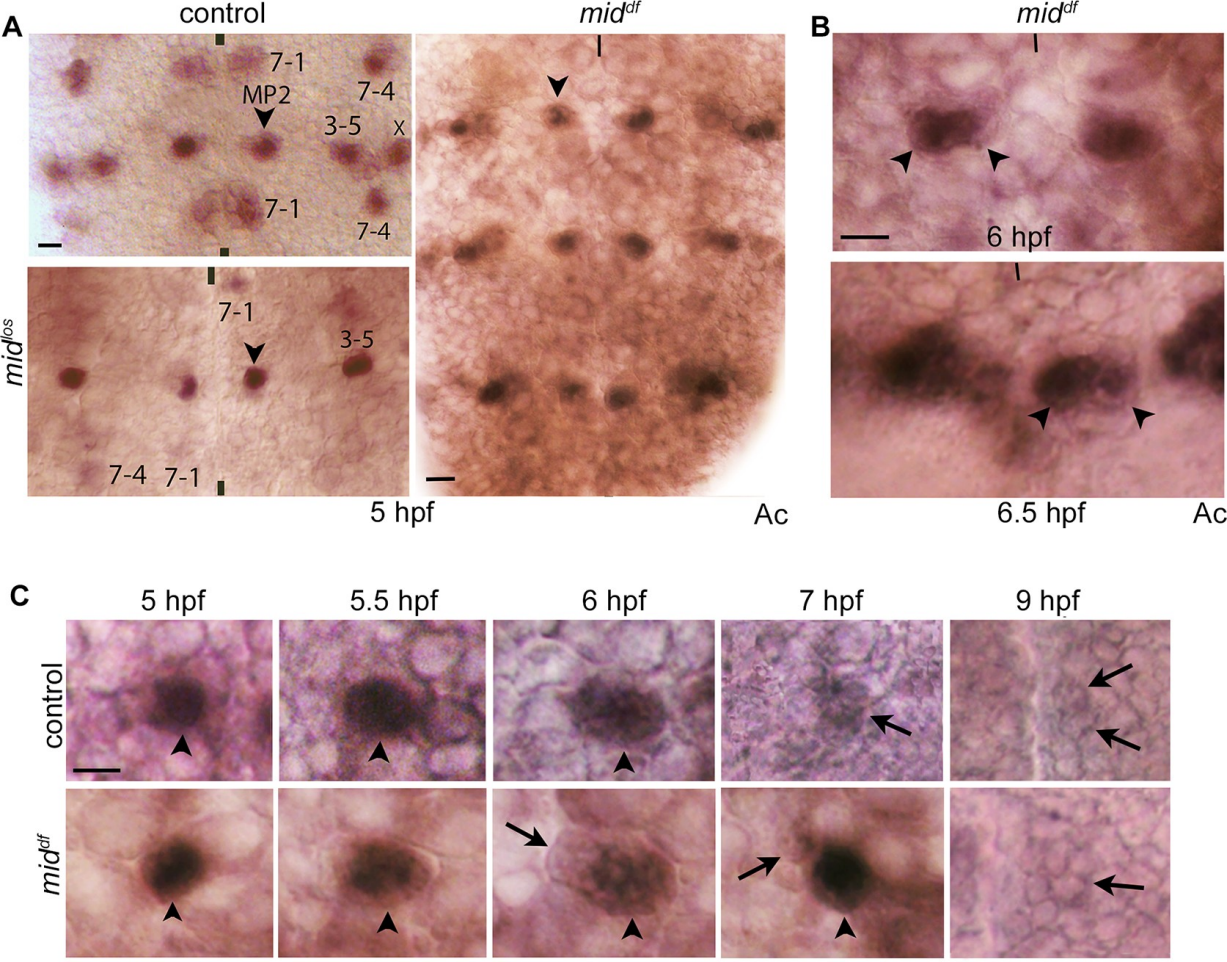
Self-renewing asymmetric division of MP2 in *mid* mutant embryos. Wild-type control and *mid* mutant embryos were stained for Ac. Ac is expressed in MP2 (and in NB3-5, NB7-1, and NB7-4) but not in its progeny neurons, v or dMP2. The anterior end is up, the midline is marked by vertical lines. See Table 1 and Supplementary Information (S1 Data) for the penetrance of the MP2 lineage defects in the mutant. Magnification bar: 10 μm. **(A, B):** In both control and *mid* mutant embryos, ~99% of the hemisegments had only one MP2 in 5 hpf embryos (A). By ~6.5 hpf, two MP2 cells were observed in a subset of the hemisegments (~4%) in the mutant (B). One or three segments are shown. **(C):** Embryos of different ages were stained for Ac. The division in 6 hpf panel shows that only the larger of the dividing cell retains Ac expression. The larger Ac-plus cell, post-division, will likely keep its identity as MP2, as suggested by the presence of an Ac-positive large cell in a 7 hpf *mid* embryo but not in the control. By 9 hpf, the cells have no Ac expression indicating that they have become progeny neurons (v/dMP2). One half-segment is shown in each panel. At least 16 hemisegments were analyzed from 4 different embryos.

Since AJ96 is expressed in MP2, but also in both d/vMP2s, we stained *mid* embryos fixed at different time-points with Achaete (Ac), which is expressed only in MP2 (Fig 2A–2C). The formation and identity specification of MP2 was normal in *mid* mutant embryos as judged by the expression of Ac with only one MP2 per hemisegment in 5 hpf mutant embryos (Fig 2A, Table 1). However, when mutant embryos that were older (~6.5 hpf) were examined, in about 4% of the hemisegments, two large Ac-positive MP2 cells next to each other were observed (Fig 2B). We examined the development of the MP2 lineage in wild-type and in *mid* mutant embryos by anti-Ac staining of a series of developmentally timed embryos. As shown in Fig 2C, in both the wild-type control and in the mutant an Ac-positive MP2 was observed in ~5 hpf. However, in the mutant, this MP2 appears to be dividing sooner than in the control (Fig 2C, ~6 hpf panels). In this asymmetrically elongated cell, the larger part had Ac-expression, whereas the other smaller part had no Ac-expression. By 7 hpf, while in the control the cell had divided with most of the AC expression in daughter cells absent, in the mutant, one of the two cells was larger and had a high level of Ac. By 9 hpf, the daughter neurons had lost Ac expression although, in the mutant, cells appeared still to retain some residual Ac. Taken together with the results from AJ96-staining (Fig 1), these results argue that MP2 undergoes a self-renewing asymmetric division in *mid* mutants.

**Fig 3:**
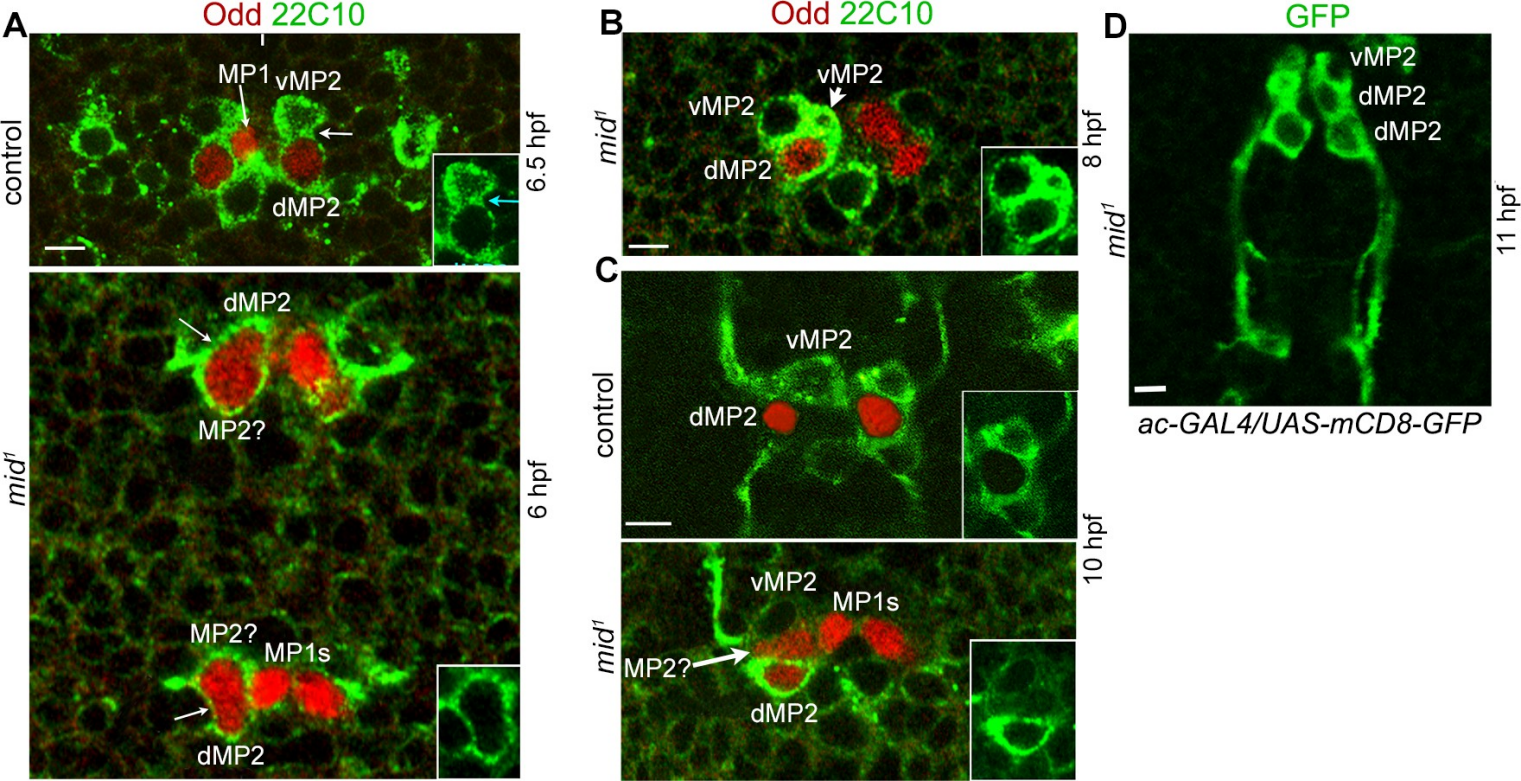
MP2 division pattern in *mid* mutant embryos. Control and *mid* mutant embryos were double-stained for Odd and 22C10 (MAP1B-like protein). Odd is present in MP2 and dMP2 but not in vMP2. MP2 starts to express 22C10 just before its division and continues to be present in v and dMP2. It reveals the cortical outline of MP2, d and vMP2 cells, and the junction between v and dMP2 has thick staining. Anterior end is up, midline is marked by vertical lines. Inset panels show only the 22C10 staining. Magnification bar: 5 μm. **(A):** A segment from a control embryo. MP2 on the left hemisegment has divided and the cytokinesis is complete with 22C10-positive thick junction between the v and dMP2 neurons. On the right, the cytokinesis is almost complete but not fully (arrow) and the thick 22C10 junction has not yet formed. Only dMP2 but not vMP2 expresses Odd. In the *mid* mutant, MP2 appears to be in mitosis but both cells have Odd, indicating that either both will become dMP2s or one will be MP2 and the other dMP2. Arrows in *mid* panels indicate the early furrow formation for cytokinesis. Since the lineage development is slightly hastened in *mid* mutant embryos (see text), *mid* embryos were examined slightly at a younger age. Two contiguous segments are shown. **(B):** A segment from a *mid* mutant embryo showing 2 vMP2 (Odd negative, 22C10 positive) and one dMP2 (Odd and 22C10 positive) neurons. **(C):** A segment from a *mid* mutant embryo showing a 22C10-positive vMP2 with its anterior projection, and a 22C10, and Odd-positive dMP2 (its posterior projection is out of the focus). This hemisegment also has a 22C10-negative, Odd positive cell, possibly an early stage MP2. **(D):** A segment from a *mid* mutant embryo showing the intersegmental fasciculation of extra dMP2 neurons. UAS-mCD8-GFP was expressed using the ac-GAL4 driver in *mid* mutant background and embryos were stained with anti-GFP.

Additional evidence for the self-renewing division came from staining *mid* mutant embryos with Odd-skipped (Odd) and Mab 22C10 (against MAP1B-like protein). Odd is expressed in MP2 and dMP2 but not in vMP2 [2]. However, 22C10 is expressed in MP2 shortly before its division and continues to be present in both v and dMP2 neurons. As shown in Fig 3A, in control, we could see an MP2 nearly completing its division (hemisegment on your right) to generate an anteriorly located and Odd-negative vMP2, and a posteriorly located Odd-positive dMP2. In the mutant, however, hemisegments where a dividing cell with one larger than the other and both expressing Odd were observed (Fig 3A, *mid*, left hemisegments). Even the nucleus appears to be larger in one of the two prospective cells (Fig 3A, lower panel). In older ~8 hpf embryos hemisegments with two vMP2s and one dMP2 could be observed (Fig 3B). We also observed hemisegments in 10 hpf mutant embryos with an Odd positive but 22C10-negative, therefore, an earlier stage MP2 (Fig 3C, arrow, left hemisegment). This hemisegment also had a vMP2 with its projection as well as a dMP2 (the projection is out of focus). These extra neurons send out their projection in the correct direction and fasciculate properly as intersegmental interneurons (Fig 3D), suggesting that these cells are unlikely mis-specified or with a “confused” identity.

**Fig 4:**
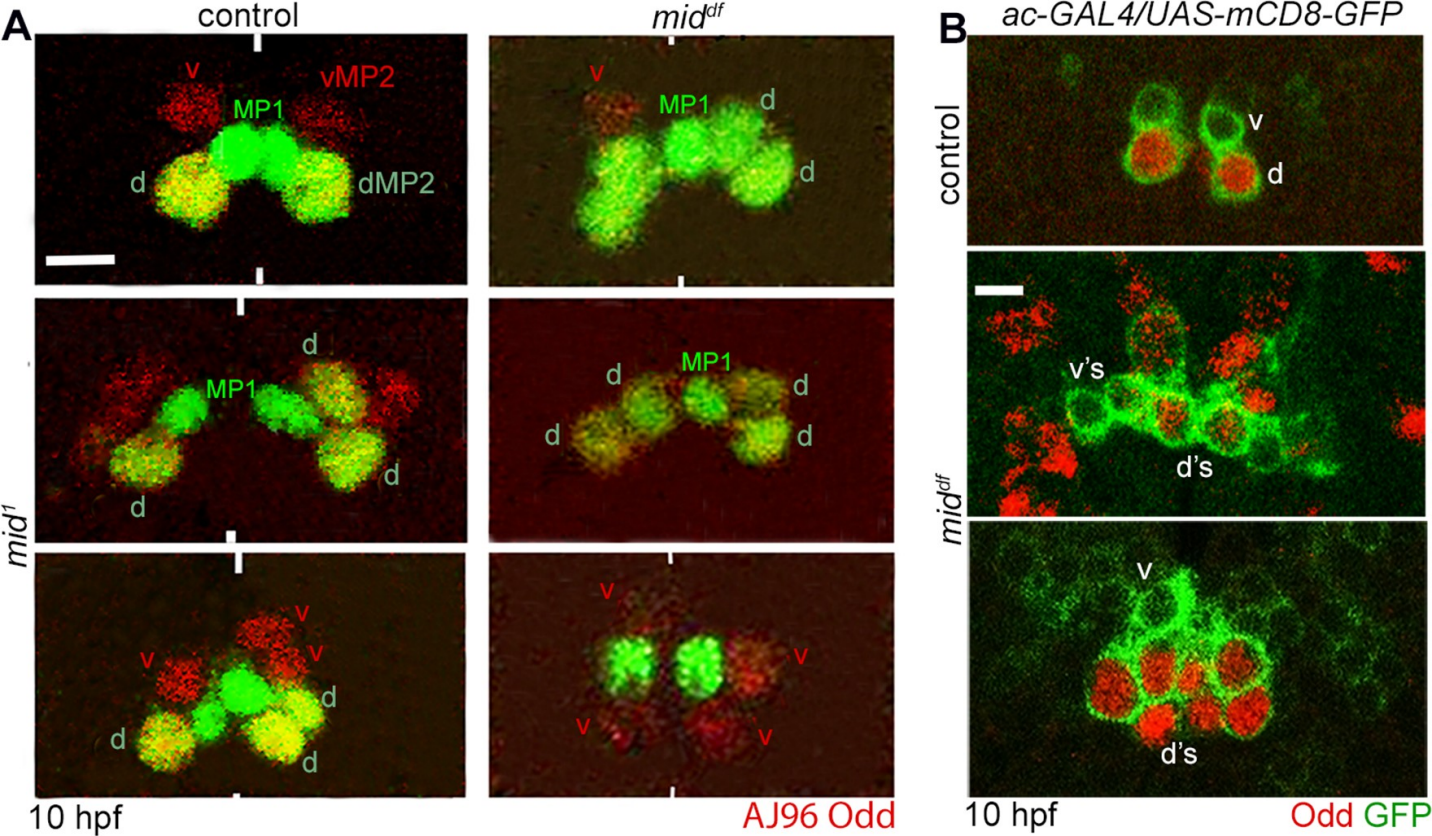
MP2 undergoes extra divisions in *mid* mutant embryos. Control and *mid* mutant embryos were double stained for AJ96 and Odd (A) or Odd and GFP (B). The anterior end is up, the midline is marked by vertical lines. See Table 1 and Supplementary Information (S1 Data) for the penetrance of the MP2 lineage defects. Magnification bar: 5 μm. **(A)**: While in control, a vMP2 (AJ and Odd-positive) and a dMP2 (only Odd-positive) were generated from MP2, in *mid* mutants (*mid*^*1*^ or *mid*, *H15*^*df*^), 2v/1d or 2d/1v or 2v/2d or 2d or 2vMP2s were generated. In hemisegments were 2 dMP2 or 2 vMP2 were formed, it was likely that MP2 symmetrically divided to generate them. Odd also stains a pair of MP1 neurons, generated from a different NB. **(B)**: Wild-type and *mid* mutant embryos expressing the mCD8-GFP (mouse CD8 and GFP fusion protein) from a *UAS-mCD8-GFP* transgene, induced in MP2 by ac-GAL4 were double-stained for Odd and GFP. Presence of multiple v and d MP2 neurons are shown.

We further examined the MP2 lineage for its division pattern with different combinations of markers. As shown in Fig 4A, hemisegments with 2 dMP2 and one vMP2, or 2 dMP2 and 2 vMP2 or 2 vMP2 and one dMP2 were observed. By inducing the UAS-mCD8-GFP transgene with ac-GAL4 driver to visualize neurons produced by MP2, multiple v and dMP2s were observed only in the mutant (Fig 4B). About 50–60 percent of the hemisegments had an MP2 phenotype in the mutant with preponderance for the formation of multiple dMP2 neurons (Table 1). It appears that in the mutant MP2 can also divide terminally and symmetrically into two dMP2s or 2 vMP2s, indicating a loss of asymmetric division (Fig 4A, middle and lower panels on your right; see also Table 1).

**Fig 5:**
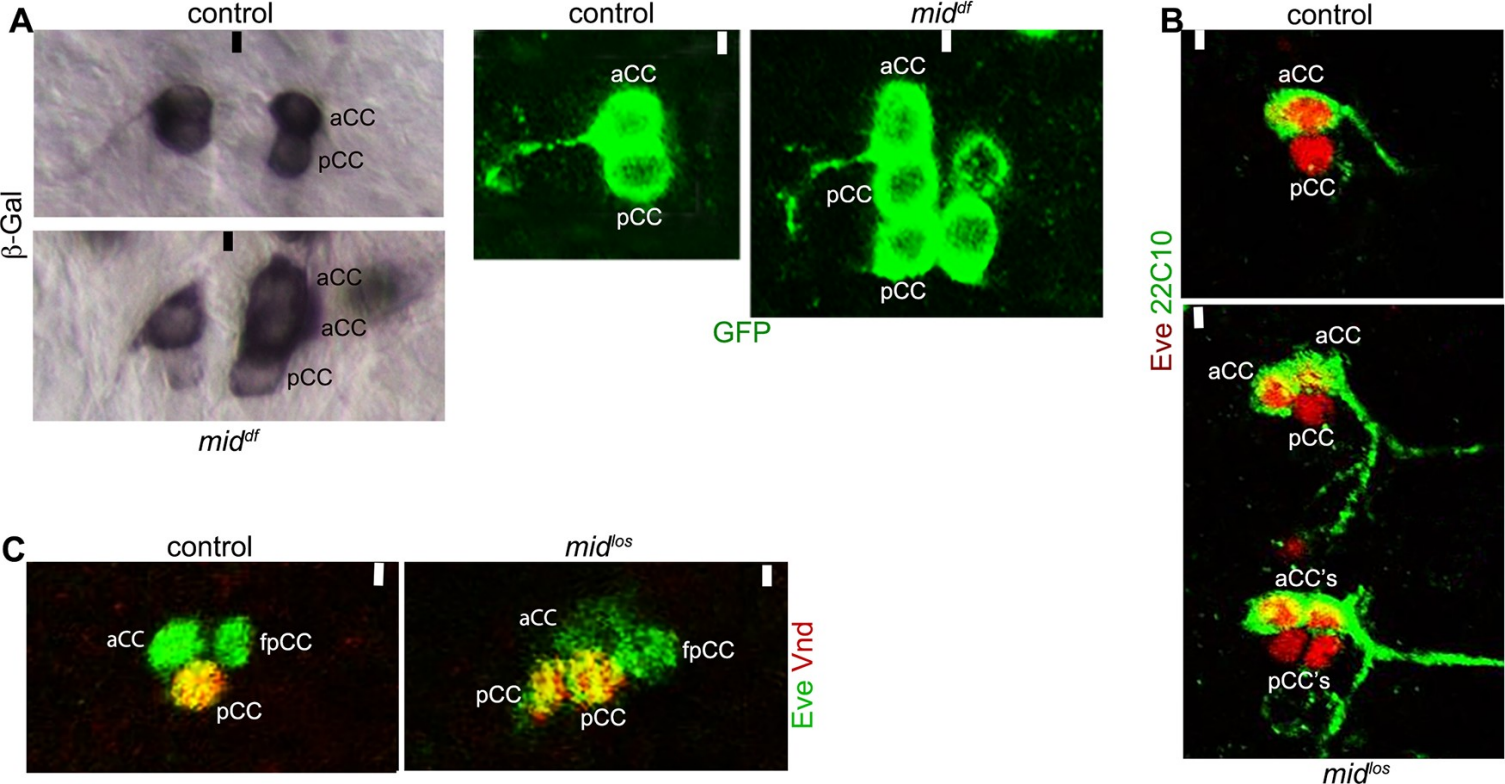
GMC1-1a of NB1-1 undergoes extra divisions in *mid* mutant embryos. About 14 hpf embryos were stained with anti-β-gal or GFP (A) or double-stained with Eve and 22C10 (B) or Eve and Vnd (Ventral nervous system defective)(C) antibodies. Anterior is up, midline is marked by vertical line. In *mid* mutant embryos, extra aCC or pCC neurons were seen in about 8% of the hemisegments (N = 330 hemisegments). **(A):** Control and *mid* mutant embryos carrying either a *UAS-tau-LacZ* (β-Gal panels) or a *UAS-tau-GFP* (GFP panels) induced with eve-GAL4 (RN2-GAL4) were examined for the aCC/pCC neurons. Shown are an aCC and pCC in the controls and either two aCCs and one pCC (A) or one aCC and two pCCs (B) in the mutant. **(B):** Control and *mid*^*los*^ embryos were examined for the aCC/pCC neurons with Eve and 22C10 staining. Hemisegments from the control with one aCC and one pCC, and from the mutant with two aCCs and one pCC, and two aCCs and two pCCs are shown. **(C):** Control and *mid*^*los*^ embryos were examined for the aCC/pCC neurons with Eve and Vnd. Hemisegments from the control with one aCC and one pCC, and from the mutant with two aCCs and two pCCs are shown. The neuron fpCC (friend of pCC) lies ventral to aCC/pCC and towards the midline and has a different projection pattern than either aCC or pCC [2]. It is also not in the same focal plane as pCC.

We also determined if *mid* affects GMC lineages since GMCs also undergo a single terminal asymmetric division to produce two post-mitotic neurons. We selected the first GMC of NB1-1 (GMC1-1a) since this lineage is well defined and its progeny neurons, aCC (anterior corner cell, a motor neuron that innervates muscle number 1) and pCC (posterior corner cell, an intersegmental interneuron), can be easily identified with different cell-specific markers [15]. As shown in Fig 5, in *mid* mutant embryos, staining with several different markers showed that while the penetrance was low, a GMC1-1a generating additional aCC and pCC neurons, was observed with preponderance for extra aCC neurons. It is likely that GMC1-1a undergoes a self-renewing asymmetric division similar to MP2, generating predominantly an aCC, but occasionally a pCC; the self-renewed GMC1-1a divides again but terminally to generate an aCC and a pCC. Since we also observed rarely two aCCs and two pCCs (Fig 5B), GMC1-1a may also divide symmetrically into two GMCs, each then generating an aCC and a pCC. These results indicate that a GMC can be susceptible to extra divisions similar to MP2 in *mid* mutant embryos.

### Prospero is cytoplasmic in MP2 in *mid* mutant embryos

**Fig 6:**
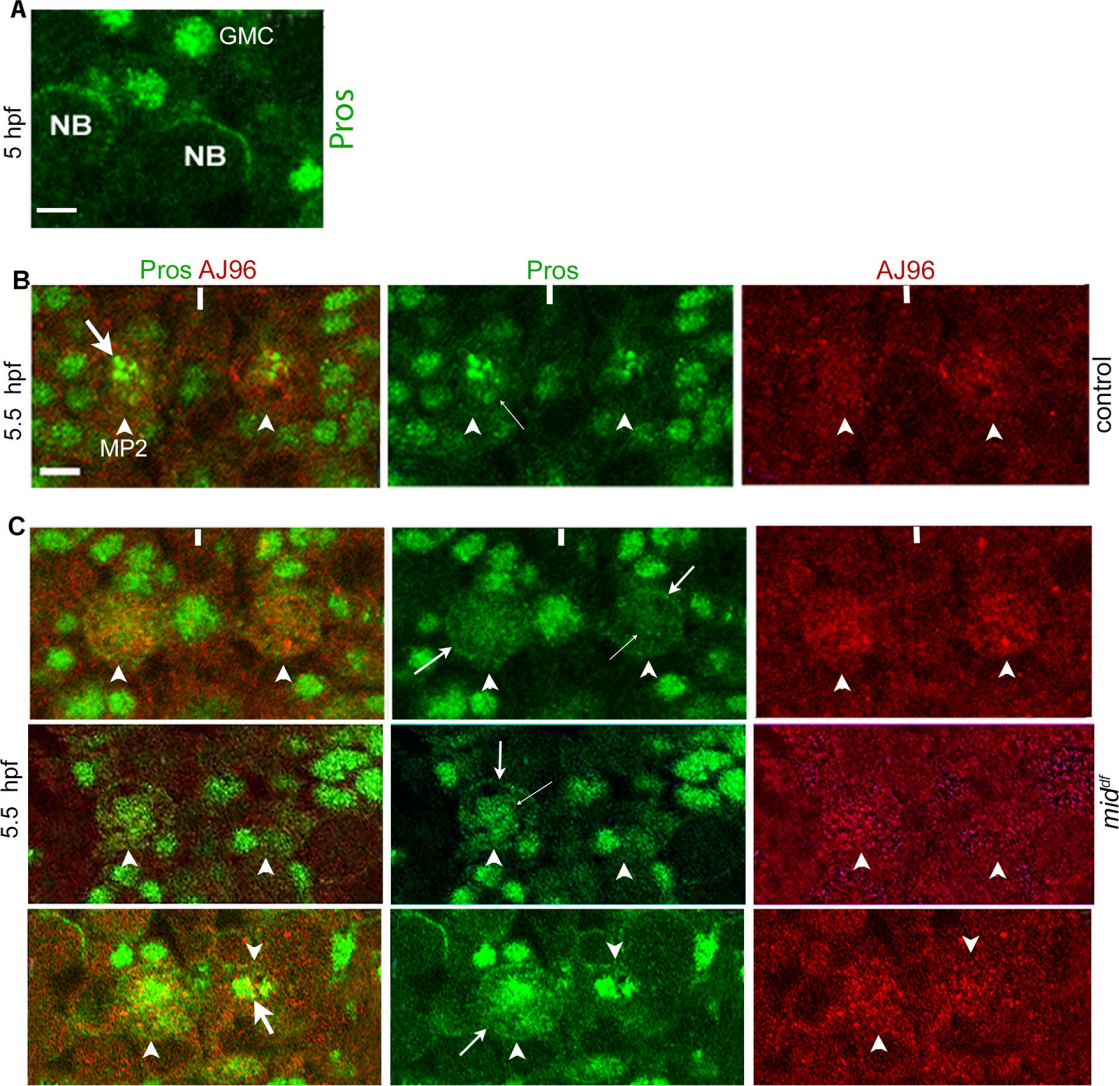
Prospero is cytoplasmic in MP2 in *mid* mutant embryos. Embryos were stained for either Pros (A) or for Pros and AJ96, an MP2 marker **(**B, C). Anterior end is up, the midline is marked by vertical lines. MP2 in panels B and C are at the same stage and are of the same size and they are not in mitosis (which occurs 6.5 hpf in wild-type and around 6 hpf in *mid*). Magnification bar: 5 μm. Arrowhead, MP2; thick arrow, chromatin Pros; arrow, non-asymmetric cortical-cytoplasmic Pros; thin arrow, nuclear Pros. **(A):** Cortical and asymmetric localization of Pros in typical NBs and nuclear localization in GMCs. **(B):** Pros localization to the nucleus/chromatin in MP2 (arrowhead) in wild-type control. Note the chromatin localization (thick arrow) within the nucleus (thin arrow), and the size of the MP2. Some cortical Pros could also be seen. **(C):** Upper row: Pros is not localized to the nucleus/chromatin in MP2 (arrowhead) in *mid* mutant embryos, instead it appears to be diffuse within the nucleus (thin arrow) and also cortical (arrow). Middle row: Hemisegments with MP2s where Pros has both cytoplasmic, non-asymmetric cortical distribution as well as in the nucleus but does not appear to be bound to chromatin. Lower row: Pros could be observed both in the nucleus and in the cytoplasm with a non-asymmetric cortical distribution (arrow); hemisegments with normal chromatin localization is also seen (thick arrow in the right hemisegment).

Previous results have shown that Prospero (Pros), a homeodomain protein with DNA-chromatin-binding activity [35], has an asymmetric and cortical localization in NBs (Fig 6A) but nuclear in GMCs (Fig 6B) [35, 36, 10] or in MP2 [10]. It has been proposed that cytoplasmic Pros NBs represents their self-renewing stem cell status, and nuclear localization signals an end to a stem cell status [37]. It is also possible that the cytoplasmic asymmetry of Pros localization is a method of segregating the protein to a committed GMC, where it becomes nuclear and functional as a DNA binding protein. Regardless, Pros localization could serve as an important marker for the self-renewing stem cell state of NBs.

In wild-type, Pros in MP2 is not only nuclear but appears to be mostly localized to the chromatin (Fig 6B, thick arrow). However, in the same age *mid* mutant embryos, Pros was not localized to chromatin, instead, it was cytoplasmic with a weak cortical distribution (Fig 6C, upper panels). This defect in Pros localization was observed in about 17% of the hemisegments (N = 300 hemisegments). We also observed MP2s where Pros was cortical and non-asymmetric as well as in the nucleus, but not on chromatin (Fig 6C, middle panels). A phenotype where Pros was cytoplasmic and cortical but also nuclear and chromatin-bound were also seen (Fig 6C, lower panels). These less severe localization defect was seen in about 11% of the hemisegments (N = 300 hemisegments). Whether this mis-localization of Pros in *mid* embryos is because of a changed division potential of MP2 or and Mid is involved in the proper localization of Pros is yet to be determined. However, these results further support the possibility that MP2s in *mid* mutants are multipotential stem cells and they undergo self-renewing asymmetric divisions similar to other NBs.

### The identity of progeny neurons generated by the self-renewing asymmetric division of MP2 in *mid* is dependent on Numb localization

**Fig 7:**
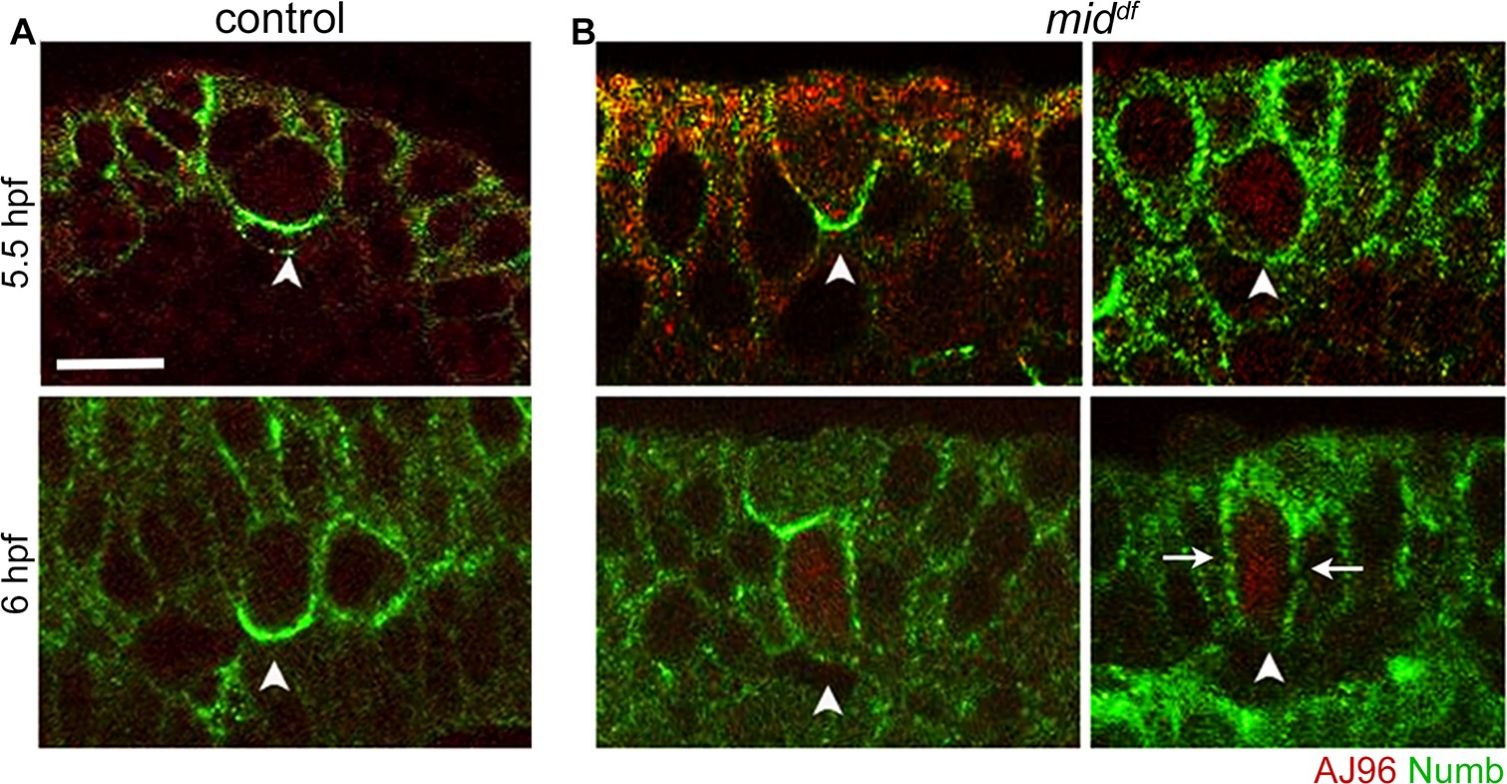
Localization of Numb is affected in *mid* mutant embryos. Embryos were stained for AJ96 and Numb. Imaging was done sideways to visualize the apical (top) and basal (bottom) distribution of Numb. See Supplementary Information for the penetrance of the defects (S2 Data). The magnification bar: 10 μm. **(A)**: Wild-type control, Numb is asymmetrically localized to the basal pole of MP2. The number of hemisegments examined (N):124 (5.5 hpf) and 84 (6.0 hpf). (**B**): In *mid* mutant embryos, MP2 with normal Numb localization as well as with non-asymmetric Numb present all along the cortex were seen. No aberrations in the plane of division or random localizations of Numb (to the apical or lateral sides) were observed. The number of hemisegments examined was 76 (for the 5.5 hpf embryos) and 57 (for the 6 hpf embryos).

Previous results have firmly established that Numb and Notch play a central antagonistic role in the asymmetric division of MP2 [7, 17]. Wherever Numb is present at high concentrations, it blocks Notch signaling from specifying a vMP2 identity, allowing the two daughters to adopt a d and a v MP2 identity. This is achieved by the localization of Numb to the basal pole of MP2 prior to its division (Fig 7A; see also S2 Data), allowing it to be inherited by only one of the two daughters. In MP2 lineage, this mechanism allows the cell with Numb to fully differentiate into a dMP2 and the other into a vMP2 identity. We determined if the localization of Numb in MP2 is affected in *mid* mutant embryos. As shown in Fig 7B, in 5 hpf *mid -*mutant embryos, 38%+/-3% of the hemisegments had a non-asymmetric localization of Numb in MP2, with Numb uniformly spread along the cortex, versus 8.6%+/-2.3% in wild-type (Fig 7A). In 6 hpf, 32%+/-4% had non-asymmetric Numb in MP2 in *mid* embryos (Fig 7B) versus 4%+/-2% in the wild-type (Fig 7A). These MP2s are expected to produce dMP2s since they will be able to block the Notch-signaling. This is consistent with the finding that in majority of the affected hemisegments, dMP2s were formed compared to vMP2s (Table 1). The MP2 cell also appears to be dividing sooner in the mutant than in the control (Fig 7B). Furthermore, the division plane of MP2 in *mid* is not randomized or disrupted, but normal as could be seen in 6 hpf embryos (Fig 7B). This result also argues that MP2 self-renews in *mid* as opposed to a transformation of other neurons or NBs into MP2. We do not know how Mid regulates the asymmetric localization of Numb, perhaps by regulating factors that mediate Numb localization.

### The expression of Mid in MP2 is highly dynamic

**Fig 8:**
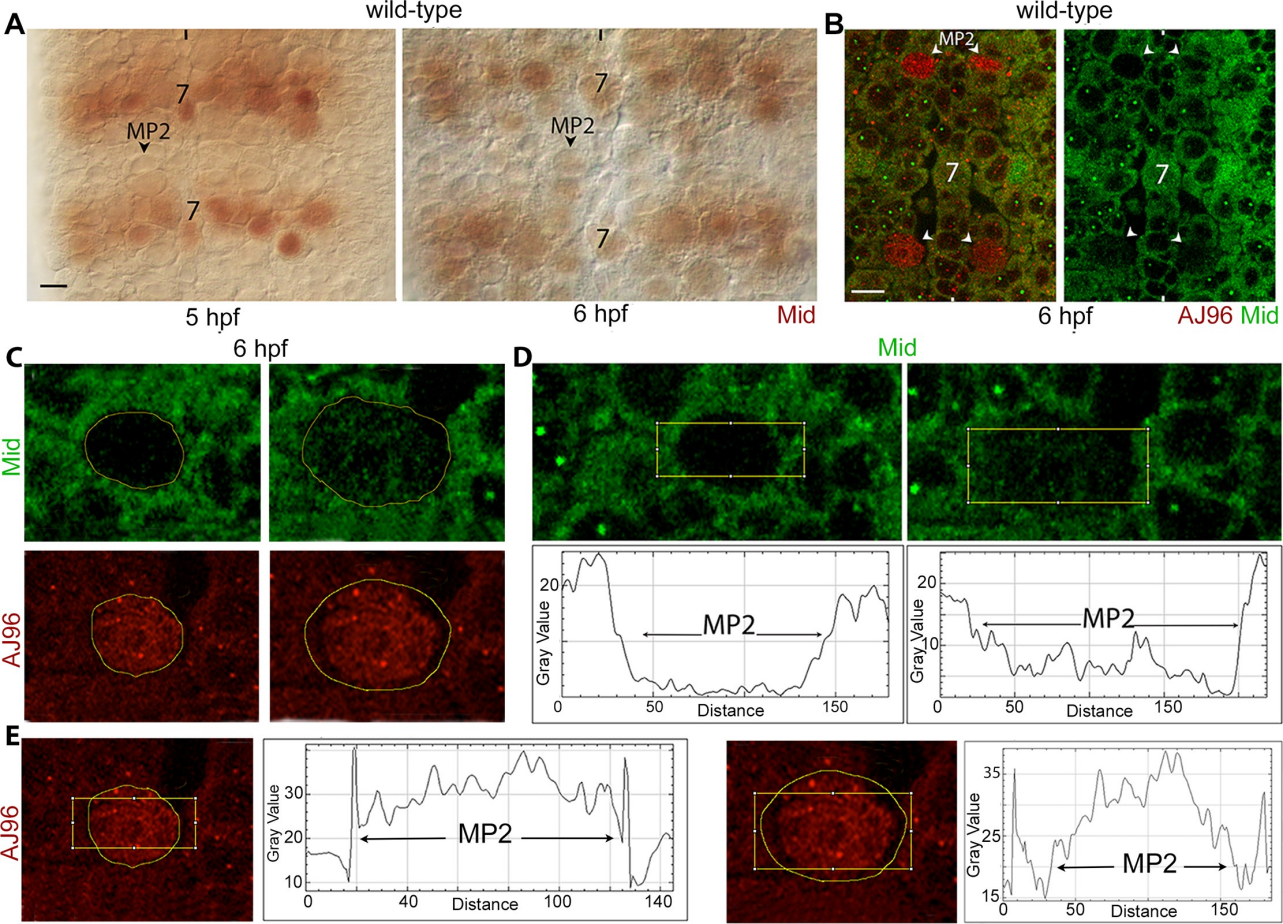
The expression pattern of Mid in MP2. The anterior end is up, the midline is marked by vertical lines. The cytoplasmic staining in Fig 8B–8D is non-specific and is seen only with confocal imaging. The magnification bar: 10 μm. ImageJ analysis was done using its plot profile function. **(A)**: Embryos were stained with Mid antibody. No discernible Mid expression could be seen in an MP2 at 5 hpf, but a faint expression of Mid is seen in MP2 in 6.0–6.5 hpf embryos. Strong expression of Mid is seen in row 7 NBs and in NB2-4. About 20 hemisegments were examined. **(B)**: Embryos were double stained for AJ96 (to identify MP2) and Mid. MP2 cells that were round and smaller had no Mid, whereas MP2s that were larger and sometimes elongated had Mid that was granular. About 20 hemisegments were examined, see also below. **(C)**: Enlarged images of two categories of MP2, the smaller MP2 lacking any appreciable amounts of Mid, and the larger one with Mid. AJ96 is a marker for MP2. **(D and E):** Distribution of Mid and AJ96 in MP2 across the cell. The ImageJ software was used to analyze the Mid and AJ96 distribution profile across MP2 using the ImageJ plot profile function. Note the granular profile of Mid in larger sized MP2. No significant differences were found between larger or smaller MP2s for AJ96 expression. At least 12 MP2s were examined, and all yielded the same patterns of Mid and AJ96 distribution.

We next examined the expression of Mid in MP2. While we did not detect any Mid protein in an early stage MP2 either in the nucleus or in the cytoplasm (Fig 8A, arrowhead, note the definition of the nuclear membrane and a thin cytoplasm), low levels of Mid were detected prior to its division (Fig 8A, compare 5 hpf and 6 hpf panels). We further examined the expression of Mid in MP2 by confocal microscopy, double-staining embryos with anti-Mid and anti-β-galactosidase (β-gal) antibody for AJ96, which identifies MP2. As shown in Fig 8B–8D, low levels of Mid was detected only in MP2s that are considered late-stage and larger in size (see also Fig 8A). Analysis of multiple MP2 cells using ImageJ tool showed two populations of MP2s in terms of Mid expression, one that are smaller and with nearly undetectable levels of Mid and the other with larger with detectable levels of Mid (Fig 8C and 8D). Our ImageJ analysis of the expression pattern of Mid in MP2s across the cells using the plot profile analytic function indicated that the larger MP2 with Mid has a punctate profile (Fig 8D). Since the loss of function for *mid* causes MP2 defects, Mid, although the level is low, it is functionally significant and necessary to prevent the self-renewal of MP2. In contrast, a similar analysis of the expression of AJ96 showed that the expression profile is the same between the smaller and larger MP2 cells (Fig 8E).

### An over-expression of Mid in MP2 blocks MP2 division

**Fig 9:**
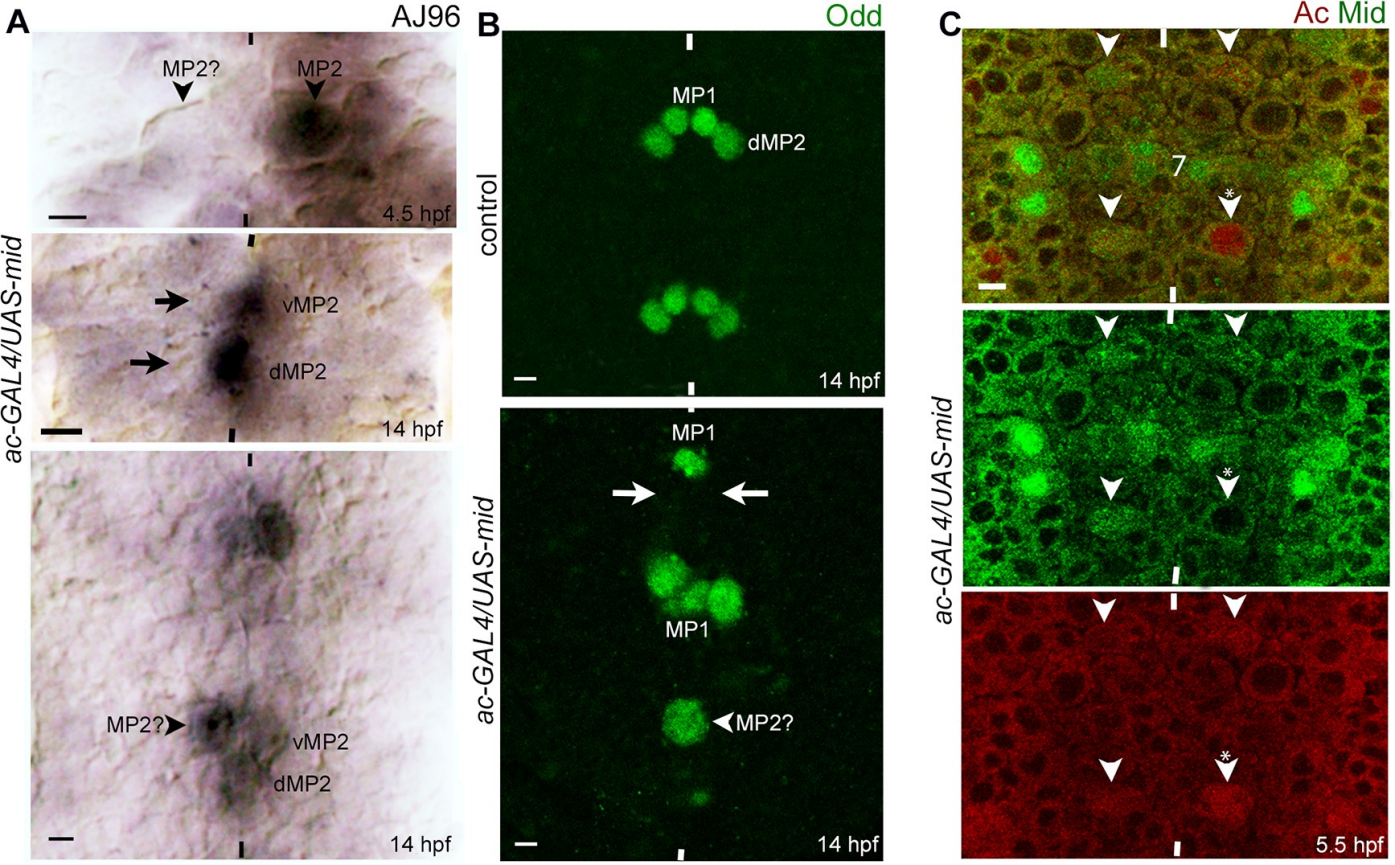
Over-expression of *mid* in MP2 affects its division pattern. A *UAS-mid* transgene was induced using the ac*-*GAL4 driver in MP2 in wild-type background. Anterior end is up, midline is marked by vertical lines. See Supplementary Information for the penetrance of the defects (S3 Data). The magnification bar: 5 μm. **(A):** The *ac-GAL4/UAS-mid* embryos were stained for AJ96, which detects MP2 as well as d and vMP2 neurons. In the upper panel (~4.5 hpf), MP2 on the right hemisgement expresses AJ96, but on the left, a NB is present in the location of MP2 but it is negative for AJ96, therefore, it likely is a mis-specified MP2. In older embryos (lower panels, 14 hpf), a hemisegment with missing v/dMP2 pairs (arrows in the upper panel) or a large AJ96-positive cell that appear to be an undivided MP2 (arrowhead) are shown. **(B):** The embryos were stained for Odd, which normally detects MP2 and dMP2 (and MP1, which is from a different NB). In *ac-GAL4/UAS-mid* embryos, hemisegments with missing dMP2s (arrows) or with cells twice the size of dMP2 (see the control panel) but the same size as an MP2 (10–12 μm) are seen. **(C):** The *ac-GAL4/UAS-mid* embryos were double stained for Ac and Mid. MP2s in hemisegments with higher than normal Mid expression (arrowhead) or low or no expression of Mid (arrowhead with a star) are shown.

To determine if increasing the levels of Mid in MP2 in wild-type leads to division defects, we induced *mid* from a *UAS-mid* transgene with ac-GAL4 and stained 5–6 hpf embryos for AJ96, or Ac. About 14 hpf embryos were also stained for AJ96 or Odd. Ac stains MP2, AJ96 stains MP2, v and dMP2, and Odd stains MP2 and dMP2 (Odd also stains MP1, a distinct lineage). Staining 5–6 hpf embryos for Ac showed that about 10% of the hemisegments were missing an MP2 (N = 6 embryos; n = 107 hemisegments; S3 Data). With AJ96, about 11% of the hemisegments were missing MP2 (N = 6; n = 99) (Fig 9A; see also S3 Data). These results suggest that over-expression of Mid in MP2 likely interferes with its identity specification. Staining of 14 hpf embryos for AJ96 showed missing v/dMP2 pairs (Fig 9A) in about 14% of the hemisegments (N = 6, n = 116; S3 Data). As shown in Fig 9B, Odd-staining of 14 hpf embryos also showed hemisegments with missing dMP2 in about 27% of the hemisegments (N = 6, n = 107) (see S3 Data). With the staining of 14 hpf embryos for AJ96 or Odd, large Odd-positive cells of about 11–12 μm size, instead of v and dMP2 neurons could be observed in 10% -11% of the hemisegments (AJ96; N = 6, n = 116; Odd; N = 6, n = 107) (Fig 9A and 9B; see S3 Data). The size of the large cell is consistent with the size of an MP2. It would appear that in these hemisegments MP2 has not divided. The missing progeny neurons in some hemisegments are likely due to misspecification of the parent MP2. Being a transcription factor, a gain of function for *mid* causing identity misspecification is not surprising as it could inappropriately suppress or activate genes and prevent identity specification of cells [20, 21].

The above results show that although the UASxGAL4 system normally induces genes at a high level, the defects in the MP2 lineage with the over-expression of *mid* using this system was limited to about quarter of the hemisegments. To examine the reason for this, we double stained embryos that are over-expressing *mid* in MP2 for Mid and Ac. Only about 50% of the MP2s had a higher than normal levels of Mid (N = 60 hemisegments), the remaining MP2s had low or undetectable levels of Mid (Fig 9C). This argues that *mid* is regulated either at the post-transcriptional or post-translational levels (or both). Nonetheless, this possibility of regulation of Mid may explain the lack of a much stronger penetrance of the phenotypes in embryos over-expressing *mid*.

### Cyclin E levels are upregulated in MP2 in *mid* mutant embryos

Given the above results, we sought to examine if *mid* affects the expression of cell-cycle genes, such as cyclins. Among the various cyclins, Cyclin E, which is a G1/S cyclin, is essential for the induction of S-phase and entry to cell cycle [29–33]. In Drosophila, Cyclin E is initially supplied as a maternal transcript. Sufficient protein is made to carry the embryo through the first 14 cleavage divisions. Subsequently, zygotic transcript is made for the next three cell division cycles. These three cycles are marked by the absence of a G1 phase: cells that exit mitosis go directly into the S phase. During these three cycles, *cyclin E* shows no cell-cycle-associated variation in transcription, but only following these three cycles [29–33].

**Fig 10:**
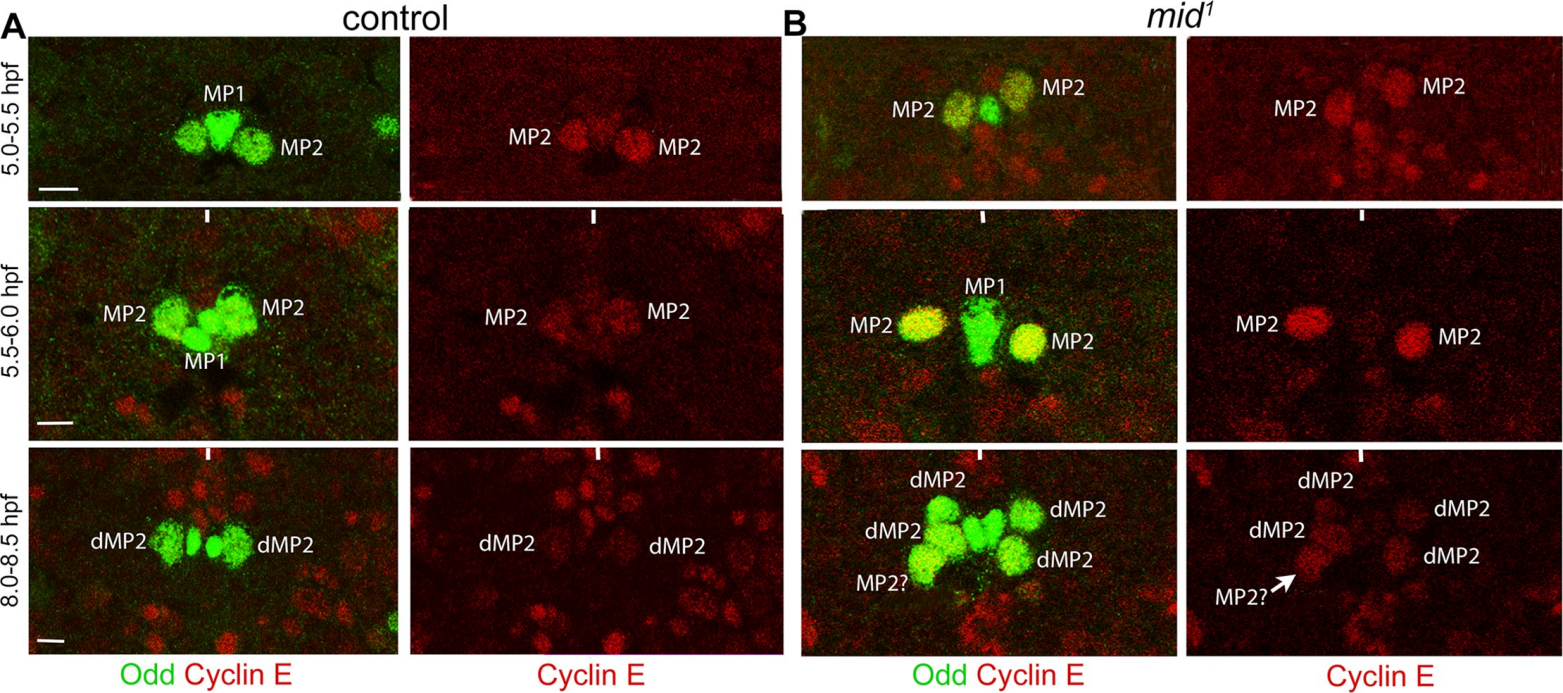
The expression of Cyclin E is upregulated in MP2 in *mid* mutant embryos. Wild-type control (A) and *mid* mutant (B) embryos were double-stained with the MP2/dMP2 marker Odd (Green; Odd stains MP1 as well) and Cyclin E (Red). Anterior is up. The image collection settings were the same for the control and *mid* embryos and between different ages. Note that in the 8–8.5 hpf *mid* panel, of the three cells in the hemisegment to the left, one cell has a higher amount of Cyclin E. It is possible that this cell will divide again. Only one segment is shown. The magnification bars: 10 μm in the 5–5.5 hpf and 5.5–6.0 hpf panels and 5 μm in the 8–8.5 hpf panels. See Fig 11 and also Supplementary Information for quantification and statistics (S4 Data and S1 Statistics).

**Fig 11:**
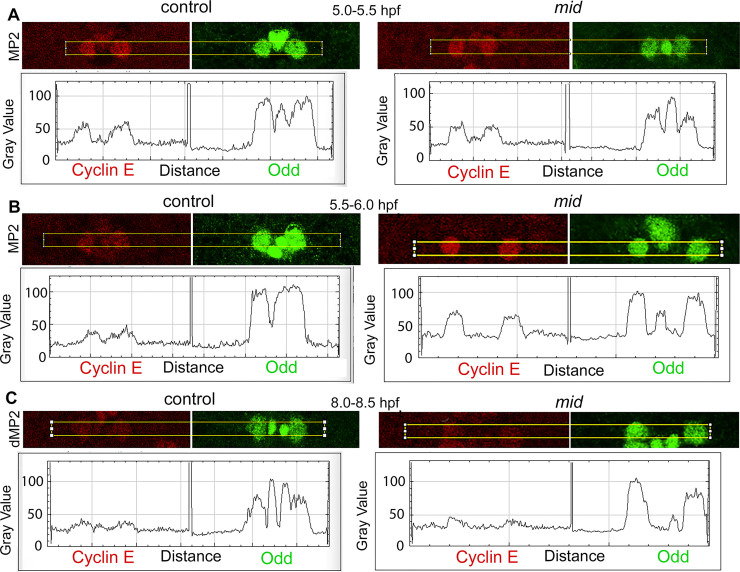
The expression of Cyclin E is upregulated in MP2 in *mid* mutant embryos. Wild-type control and *mid* mutant embryos were double-stained with the MP2/dMP2 marker Odd (Green) and Cyclin E (Red). Anterior is up. The image collection settings were the same for the control and *mid* embryos and between different ages. The ImageJ analysis was done using its plot profile function. The image resolution (300 Pixels/Inch) and pixels (3660 Pixels) were exactly the same for all images. Two-Sample T-Test or Welch’s T-Test was used to test statistical significance between the control and the mutant in Cyclin E and Odd expression in three different ages (see S5 Data). The difference in the expression of Cyclin E in MP2 between wild-type and *mid* at 5.0–5.5 hpf window (N = 6) was not statistically significant with a P = 0.631777. The difference in the expression of Odd for the same window (N = 6) was significant with a P = 0.0146960. The difference in the expression of Cyclin E in MP2 between wild-type and *mid* at 5.5–6.0 hpf window (N = 6) was statistically significant with a P = 5.61231e^-7^. The difference in the expression of Odd for the same window (N = 6) was not significant with a P = 0.285161. The difference in the expression of Cyclin E between wild-type and *mid* (N = 6) at 8.0–8.5 hpf was not significant with a P = 0.679337. For Odd however, the difference at this window was significant (N = 6) with a P = 0. 00496460. See text and Supplementary Information for additional statistical treatments between time-points within groups and between groups (S4 Data and S1 Statistics).

Since one of the progenies of the MP2 division stays within the cell cycle in *mid* mutants, we examined the levels of Cyclin E in MP2. In wild-type, MP2 is formed around 4 hpf and it divides around 6.5 hpf. We stained wild-type control and *mid* mutant embryos that are 5.0–5.5 hpf, 5.5–6.0 hpf and 8.0–8.5 hpf with Cyclin E and Odd (Odd is to identify MP2 and dMP2). As shown in Fig 10A and 10B, the levels of Cyclin E in MP2 in 5.0–5.5 hpf embryos were about the same between wild-type and *mid* mutant embryos. Statistical analysis of the plot profile of the expression showed no statistically significant difference between the two (Fig 11A; S4 Data, S1 Statistics). Interestingly, there was a statistically significant difference in the expression of Odd between the two with the control having a higher level of Odd. Odd in wild-type embryos is not expressed in a newly formed MP2, but it can be detected after about 30 min of its formation. The lower levels of Odd in the *mid* mutant suggest that MP2 in the mutant may have retained certain properties of an early MP2. In 5.5–6.0 hpf embryos, the expression of Cyclin E in control is down regulated (Fig 10A; compare Fig 11A and 11B) and this down-regulation was statistically significant (S4 Data, S1 Statistics). However, the level of Cyclin E in MP2 in the mutant was increased in 5.5–6.0 hpf embryos, and this increase was statistically significant compared to the mutant at 5.0–5.5 hpf or to the wild-type control in both developmental stages (Figs 10B and 11B; S4 Data, S1 Statistics). The difference in the levels of Odd between the control and the mutant MP2 was minimal with no statistical significance (S4 Data, S1 Statistics). In post-mitotic dMP2 (and presumably also in vMP2), the level of Cyclin E was further down regulated in wild-type control (8.0–8.5 hpf panel, Figs 10A and 11C). A similar down regulation was also observed in *mid* mutant embryos, and this down-regulation was statistically significant compared to the 5.5–6.0 hpf mutant MP2s, but the difference in Cyclin E levels between the wild-type and the mutant dMP2 was not significant (Figs 10 and 11C; S4 Data, S1 Statistics). However, there was a statistically significant difference between the wild-type and the mutant in Odd expression with Odd being higher in the mutant (Figs 10, 11C, S4 Data, S1 Statistics). We have not investigated the significance of this dynamic Odd expression. Additional statistical comparisons of the levels between different time points within the group (wild-type control and *mid*) or between the groups of different time-points are given in Supplementary Information (S1 Statistics). In summary, these results show that there is an upregulation of Cyclin E in MP2 in *mid* mutants and argue that Mid functions as a repressor of *cyclin E* [see also ref. 20].

### Mid binds to the TBE site in *cyclin E* promoter

**Fig 12:**
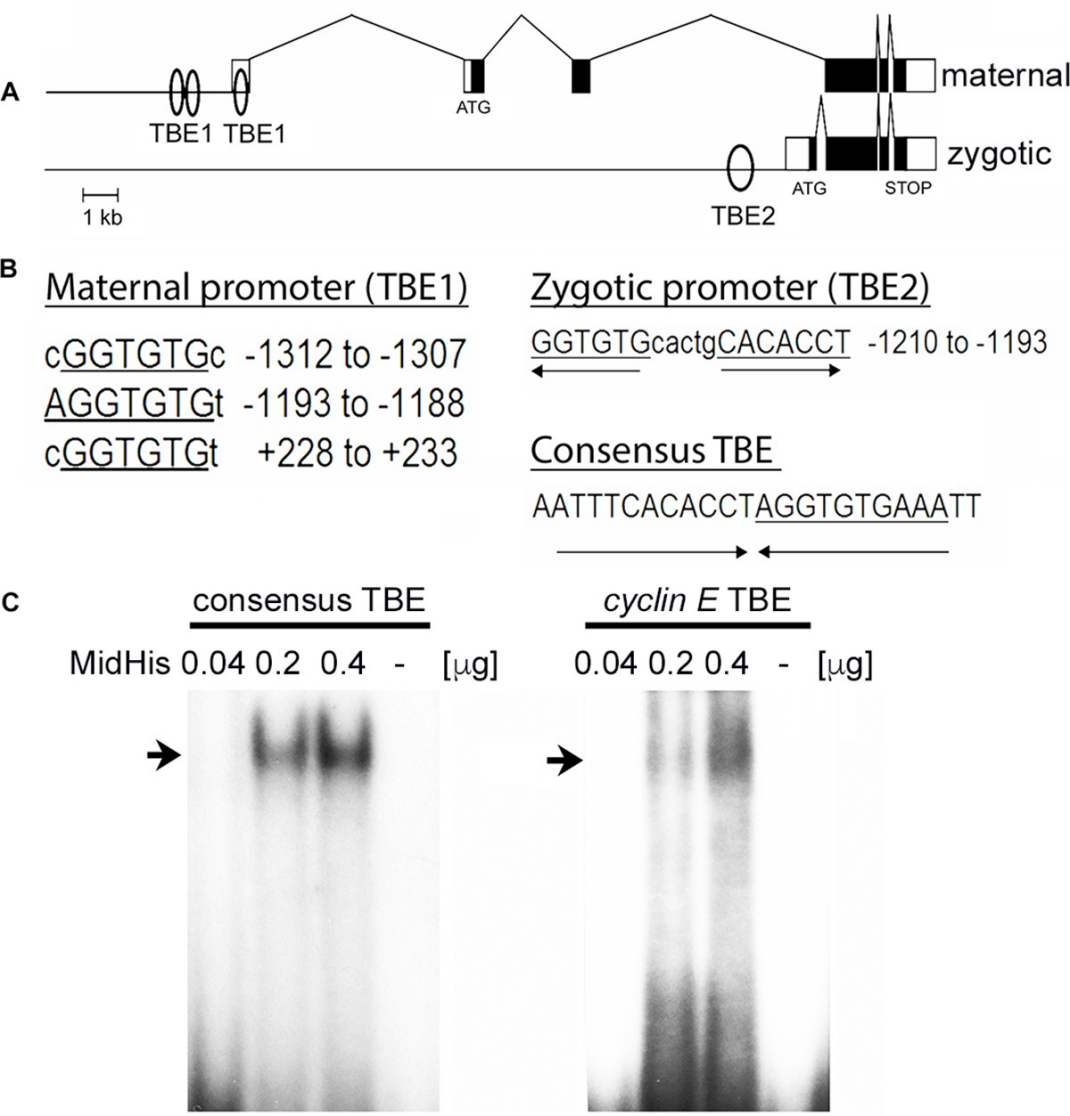
Mid binds to a variant TBE present in the *cyclin E* promoter. **(A):** TBE in the maternal and zygotic *cyclin E* promoter. TBE1 (2 elements) is in the maternal promoter and TBE2 is in the zygotic promoter. **(B):** TBE1 and TBE2 DNA sequence of *cyclin E* promoters and the consensus TBE sequence. **(C)**: Gel shift assay, showing the binding of Mid (arrow) to the *cyclin E* TBE2 labeled with P-32. The zygotic TBE2 is similar to the ideal TBE consensus sequence of the *brachyury* gene (in which the TBE was first identified), except that the TBE2 of the *cyclin E* promoter is an inverted palindrome (we have named this a variant TBE). Note that the binding to the consensus sequence is tight and the band is compact, whereas the band is less compact with the *cyclin E* TBE2. The bands represent P32-labeled DNA probes bound to Mid (His-tagged Mid).

We sought to explore the possibility that Mid represses *cyclin E* via binding to its promoter. This would require the presence of Mid-binding TBE in the promoter of *cyclin E*. The maternal promoter of *cyclin E* has two TBE sites and the zygotic promoter has one (Fig 12A and 12B). We focused on the zygotic promoter and since this TBE has the two parts of the consensus sequence running in opposite direction (Fig 12B), we determined if Mid can bind to this TBE using the gel shift assay. Purified Mid protein was incubated with P-32 labeled consensus TBE-sequence or the variant TBE sequence of the *cyclin E* zygotic promoter and the gel-shift assay was performed. As shown in Fig 12C, Mid binds the consensus TBE and the binding was dose-dependent; the Mid protein was also able to bind the *cyclin E* promoter-TBE, perhaps not as efficiently as the consensus site (a compact band with the consensus TBE versus a less compact or diffused band with the *cyclin E* TBE). Nonetheless, the ability of Mid to bind to the *cyclin E* TBE, taken together with the result that in *mid* embryos there is a de-repression of *cyclin E* expression (Fig 10), argues that Mid indeed represses *cyclin E* in MP2 via binding to its promoter, but this repression may not be necessarily complete as an ON/OFF switch (see Discussion).

### A gain of function for *cyclin E* in MP2 causes the same self-renewing phenotype as loss of function for *mid*

**Fig 13:**
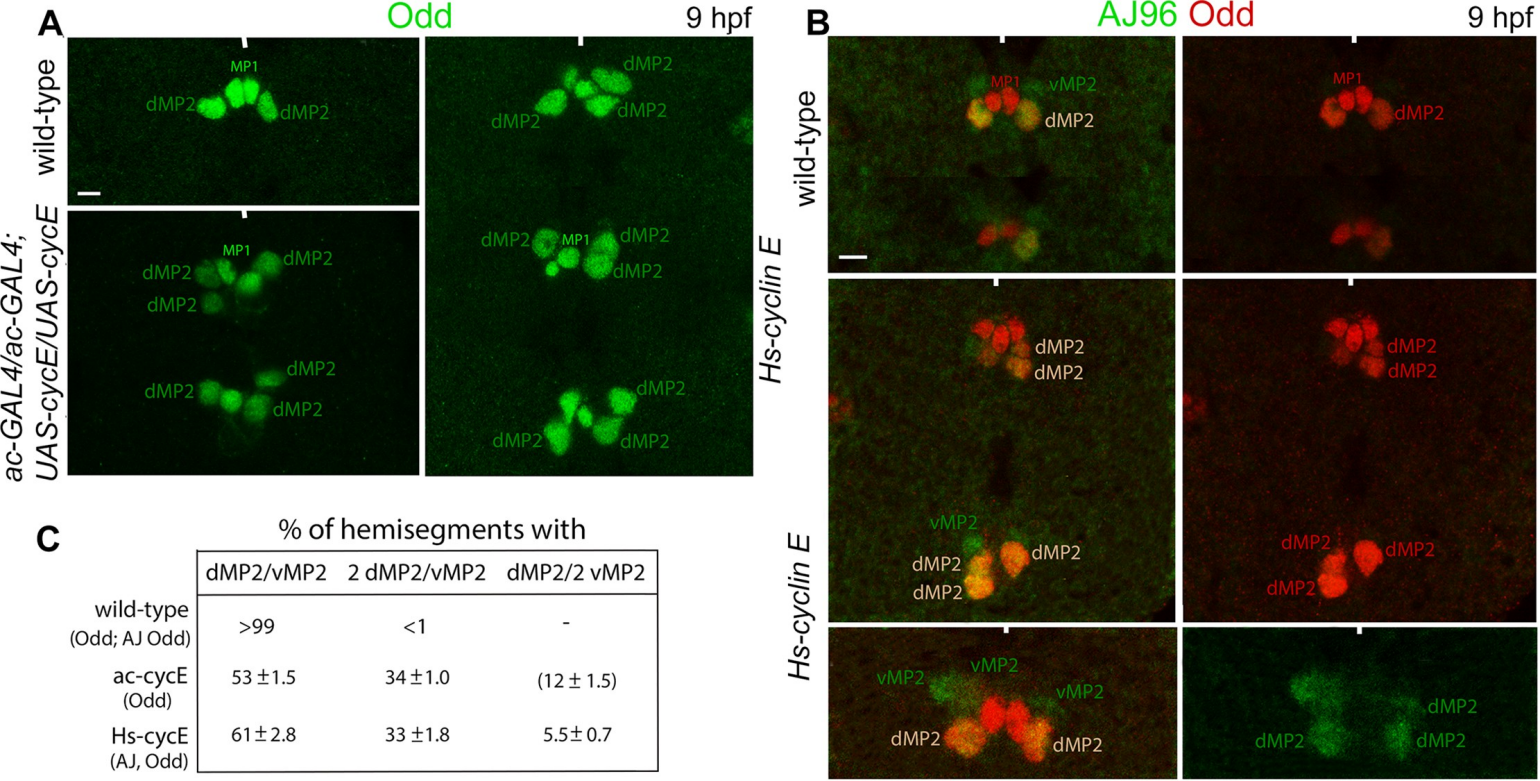
The over-expression of *cyclin E* in MP2 generates additional d and vMP2 neurons as in *mid* mutants. The *cyclin E* gene was induced in MP2 from a *UAS-cyclin E* transgene using the ac-GAL4 driver (A) or by a brief heat shock of embryos carrying an Hsp70-promoter linked *cyclin E* transgene (*Hs-cycE*) (B). These embryos were stained for Odd, which identifies dMP2 at this stage of development (A) or AJ96 and Odd, where AJ96 identifies both v and dMP2, and Odd stains only the dMP2 (B). The anterior end is up, the midline is marked by vertical lines. One to three segments are shown. See Supplementary Information for the penetrance of the defects (S5 Data). The magnification bar: 5 μm. **(A and B)**: Additional dMP2s (A) or d and vMP2 (B) with the over-expression of *cyclin E* either from *ac-GAL4xUAS-cyclin E* or *Hs-cyclin E* are shown. **(C)**: Penetrance of the phenotype(s), note the preponderance for the formation of dMP2s as in *mid* mutant embryos. The numbers within the bracket is based on calculation: 100- (normal + defective hemisegments) (See S5 Data).

Our results suggest that loss of function for *mid* results in the de-repression of *cyclin E*, which in turn maintains one or more of the progenies of the initial MP2 division within the cell cycle. In this scenario, ectopic expression of *cyclin E* in MP2 outside of the control of Mid should also result in the self-renewal of MP2. First, we induced the *UAS-cyclin E* transgene in MP2 in wild-type embryos using the MP2-specific ac-GAL4 driver. These embryos, aged about 9 hpf, were examined with Odd staining. As shown in Fig 13A (see also S5 Data), the over-expression *cyclin E* in MP2 generated additional dMP2 neurons. Second, we also induced *cyclin E* using a transgenic line carrying the *cyclin E* gene linked to the *heat shock protein 70* gene promoter (*Hs-cyc E*). The transgene was induced briefly in embryos that are 5–7 hpf. After aging for another 3–4 hours, the d and vMP2 neurons were examined by staining these embryos for AJ96 and Odd (Fig 13B). As shown in Fig 10B (see also S5 Data), this induction resulted in the generation of extra d and vMP2 neurons. These phenotypes were strikingly similar to those produced by the loss of function for *mid*. Moreover, as in loss of function for *mid*, over-expression of *cyclin E* also produced predominantly extra dMP2 neurons (Fig 13C), indicating that the preference for dMP2 formation with the self-renewing asymmetric division of MP2 is common to both situations.

## Discussion

There are very few cases in which we understand the control of the entry and exit of cells from the cell cycle. It can be argued that these controls are immensely important in the development of the nervous system, which is executed by stereotyped lineages coupled to regimented changes in developmentally important genetic players. Our data indicate that *mid* is one of the genes that may regulate entry/exit from the cell cycle of neural stem cells—to divide or not to divide or when to divide. Thus, *mid* may provide a much-needed entry point to understand these immensely important processes by tying the division potential of precursors to *cyclin E*, ultimately determining a certain number of neurons are produced from precursor cells during neurogenesis. Several previous studies have shown that *mid* plays a significant role in development and disease from flies to humans [19–21, 23–27, 38]. The results described in this paper add to the list of roles played by this important gene.

**Fig 14:**
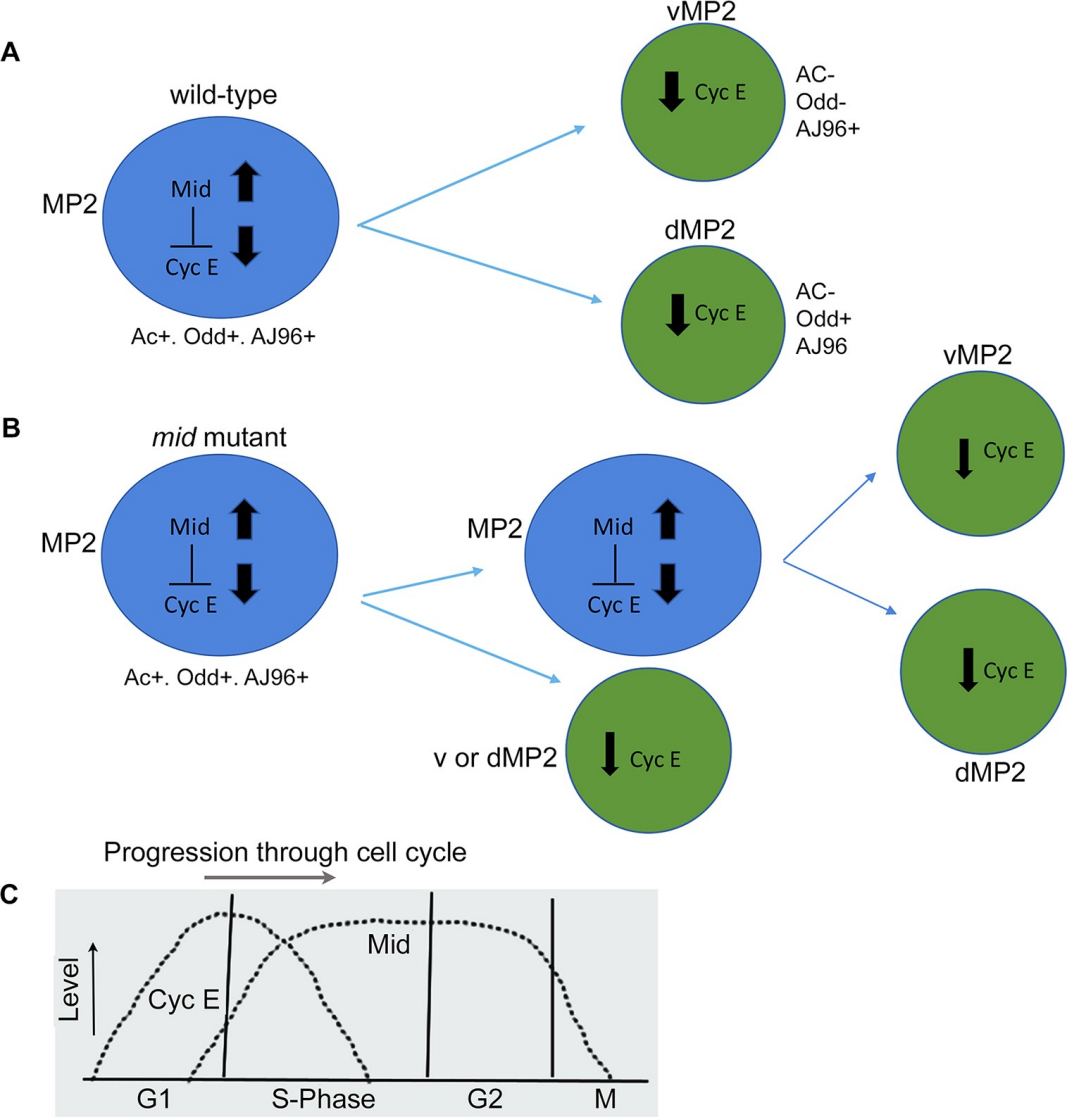
MP2 lineage development in wild-type and *mid* mutant embryos. Line drawings showing the developmental history of MP2 in wild-type and *mid* mutant embryos. Ac is Achaete, which is specific to MP2; Odd is present in MP2 and dMP2, and AJ96 is expressed in MP2, v and dMP2 neurons. (**A**): In wild-type, MP2 expresses Ac, Odd, AJ96 and Mid (low levels). It divides to generate the anteriorly located vMP2, which expresses AJ96 but not Ac or Odd, and projects its axon anteriorly, and the posteriorly located dMP2, which expresses Odd and AJ96 but not Ac, it projects its axon posteriorly. Mid is upregulated and Cyclin E is downregulated in MP2 prior to division. (**B**): In the mutant, MP2 expresses Ac, Odd, and AJ96 as in wild-type control but it divides to generate a larger cell that retains Ac, Odd and AJ96 expression as in a MP2. The second cell is smaller and is negative for Ac and more frequently has Odd and AJ96 expression consistent with its identity as a dMP2, and less frequently has AJ96 expression but not Odd or Ac expression, consistent with a vMP2 identity. In older embryos, hemisegments with a 3-cell phenotype is generally observed although a 2 cell or multicell hemisegments are also seen. **(C):** Line drawing to illustrate the levels of Cyclin E and Mid in MP2. In the absence of *mid* function, Cyclin E level is maintained until mitosis and often high level is inherited by one or more progeny cells.

We argue that Mid mediates precursor cell exit from the cell cycle by regulating in part *cyclin E* (summarized in Fig 14). How does Mid do this? A critical amount of Cyclin E is essential for the cells to go from G1 (or G0) to S-phase [30–33]. Cyclin E level is down-regulated towards the end of S-phase. In MP2, this down-regulation of Cyclin E likely coincides with the up-regulation of Mid since Mid is present only at a later part of the MP2 cell cycle (Fig 14C). In *mid* mutant embryos, Cyclin E is not down regulated in MP2; *cyclin E* promoter has Mid-binding TBE and Mid binds to this TBE. These results argue that Mid acts as a repressor of *cyclin E*, which is also consistent with the finding that loss of function for *mid* produces the same MP2 phenotype as gain of function for *cyclin E*. One caveat is that when MP2 divides, the progeny has very little detectable Mid. How is the repression of *cyclin E* maintained in these cells? It may be that some other gene takes over the role of Mid, or that Mid has set up a chromatin “state” during the M-phase and that “state” guides the cell(s) to exit the cycle and become post-mitotic. Regardless, our results show that an inter-play between Mid and Cyclin E is part of the cellular strategy that keeps the progeny of MP2 from re-entering the cell-cycle. We want to point out that a gain of function for *cyclin E* in GMCs also induces GMCs to undergo self-renewing asymmetric divisions in the same fashion as MP2 does [39].

In *mid* mutant embryos, and likely also in *cyclin E* gain of function embryos, when MP2 divides, whichever cell randomly has a higher level of Cyclin E appears to maintain the MP2-like state and re-enters the cell cycle. The cell that has a lower level of Cyclin E becomes committed to a differentiation pathway (Fig 14). If both the progeny have higher levels of Cyclin E, which is above the threshold required to enter cell cycle, we think that both will retain the MP2 identity for at least one additional division, resulting in a 2 d and 2 vMP2 phenotype (Fig 4A). We do not know if an asymmetric inheritance of Cyclin E occurs during division, or in one of the daughters Cyclin E is selectively degraded in line with its differentiation state or future. A similar situation exists for Odd expression in MP2 lineage as well. When MP2 divides, Odd is either not segregated to the presumptive vMP2, or it gets very quickly degraded in that cell (Fig 3A).

A loss of function for *mid* up regulates Cyclin E, but it also causes the retention of MP2 identity in one of the daughters of MP2. This daughter retains a strong, MP2-like Ac expression and is also larger similar to an MP2. Thus, this is not simply a matter of moving through the offspring’s G1 checkpoint. Since Mid is known to have a role in identity specification [18, 20, 21], its expression in late MP2 may be involved not only in the down regulation of *cyclin E* but also to prevent its self-renewal as an MP2. This is further supported by the finding that in *mid* mutants, Pros in MP2 does not remain localized to chromatin in the nucleus but is cytoplasmic similar to other NB stem cells (Fig 6). Furthermore, Numb localization in MP2 in the mutant is non-asymmetric (Fig 7). It may be that non-nuclear/chromatin Pros and non-asymmetric Numb plays a role in conferring a self-renewing capability to MP2 in *mid* mutants. Mid might also regulate genes necessary for Pros and Numb localization, or these affects might be a consequence of the change in MP2 division potential. We would also note that an upregulation of Cyclin E in *mid* mutants might itself mediate the retention of MP2 identity by one of the daughters since over-expression of *cyclin E* outside its normal controls leads to the same MP2 lineage phenotype as loss of function for *mid*. The significance of our results described here extend beyond the generation of a few extra neurons in one NB lineage and one GMC lineage, it provides a window as to how a large number of neurons in specific lineages or regions would have been generated during evolution, a process by which humans acquired a significantly larger neocortex compared to our immediate relatives in the evolutionary tree.

### Regulating the number of cell divisions of precursor cells

It is not known which genes or mechanisms restrict the division potential of NBs or GMCs. The same mechanism may operate in these precursor cells to limit their division potential. Mid appears to be a player in this process. One of the questions is whether the mechanism involves an elapsed developmental period (time-elapsed, and unidirectional), or is it the number of divisions a parent cell counts. The elapsed-time model will require a sequential and time-sensitive build-up of regulators/events that are independent of cell division. The cell division counting mechanism will rely on the number of cell divisions that occurs for events or gene expression changes to take place. There is evidence that events and gene expressions do not strictly follow elapsed time but dependent on cell division [40, 41]. The Drosophila embryonic nerve cord is organized into hemisegments, and each hemisegment is more or less the same as the other with the same set of neurons. But there is heterogeneity in the timing of cell division of the same lineage between hemisegments. The gene expression also varies accordingly between hemisegments. Thus, unless the measurement of the time itself varies between cells/lineages (or hemisegments), there may not be a precise measuring of time-elapsed that regulates events and gene expression programs. The gene expression program in a NB changes over time, and this change is NB-division-dependent [40, 41]. We also think that there may be a unidirectional, elapsed time mechanism with some plasticity, and it may interact with the division-counting mechanism to prevent an uncontrolled cell division of precursor cells.

### Mid-regulation of asymmetric division of precursor cells

Mid appears to affect both the self-renewing asymmetric division as well as the terminal asymmetric division. For example, MP2 that self-renews in *mid* mutants also generates extra post-mitotic neurons, whose identity is dependent on the localization of determinants such as Numb (Figs 1–4). We do not believe these extra neurons are generated from a second NB that has adopted an MP2 identity (or from the MP2-equivalence group, as in neurogenic mutants) since mostly one MP2 is seen in the mutant (Figs 1C, 2A and 2C). We did find rarely two MP2s (Fig 2B), but they appear to be from the symmetrical division of MP2 into two MP2s. Furthermore, a duplication of MP2 due to the transformation of another NB to MP2 or a second MP2 forming from the equivalence group would generate even-numbered progeny neurons. In *mid*, the majority of hemisegments generate odd-numbered neurons (Figs 1–4; Table 1). The cytoplasmic Pros localization in MP2 in *mid* (Fig 6C) certainly confirms that MP2 has adopted a NB stem cell identity. These arguments also apply to GMC lineages, where we found, for example, GMC1-1a generating odd numbered progeny neurons (Fig 5). In how many NB/GMC lineages Mid regulates the division potential is not known, but based on the expression pattern, we think that the division of a subset of NBs/GMCs is subjected to Mid regulation. Finally, a transformation of epithelial cells to neurons in *mid* is not likely since we do not see holes in the cuticle [18, 21]. The partial penetrance of the defects in *mid* mutants might be arising from a partial redundancy for the pathway or more likely the *mid* gene since the fly genome has about 8 known Tbx genes.

Loss of function for *mid* can affect NB identity [21]. Is it possible that MP2 adopts a different NB identity in *mid* mutants? The answer is unlikely since MP2 in the mutant expresses markers that are specific for MP2 and not for other NBs. They also produce progeny neurons specific to the lineage. For the same reason, we do not believe an MP2 or its progeny in *mid* embryos are “confused”. A parent cell that has confused or rather has an altered identity will produce neurons specific to the identity that the MP2 has transformed into, or a new identity from a confused state.

The role of Mid in the terminal asymmetric division, i.e., what will be the identity of the progeny neuron, appears to be tied to the asymmetric segregation of Numb. Previous results have shown that Numb segregates to the basal pole of MP2 and when MP2 divides, only the presumptive dMP2 inherits Numb [7]. The function of Numb is to block Notch signaling from specifying a vMP2 identity [7] and Notch signaling appears to begin acting on MP2 cell itself before its division [17]. In *mid* mutants, Numb is frequently seen non-localized and distributed throughout the cortex of MP2 (Fig 7B) instead of its normal basal pole localization (Fig 7A), As a result, one can observe a dividing MP2 with both cells inheriting Numb in *mid* mutants. In such hemisegments, asymmetric self-renewal of MP2 likely produces a dMP2. But, in those hemisegments that produce vMP2s, we suspect Numb localization is asymmetric or absent. In either case, the apical/basal plane of division of MP2 does not seem to be affected (Fig 7B). Additionally, a non-localization of Numb has not been associated with randomization or disruption of plane of division, it only causes progeny neuronal identity switch, or identity misspecification [see ref. 7, for example]. We think that *mid* affects Numb localization indirectly. Being a transcription factor, it could alter the expression of any of the genes that are part of the Numb-localization mechanism. It could also affect *numb* expression directly, albeit in a partially redundant manner.

### Mid and Cyclin E relationship in MP2

The entire regulation of Cyclin E by Mid is intriguing. For one thing, evolution appears to design mutant or variant TBE sequence in the *cyclin E* promoter, especially in the zygotic promoter of *cyclin E*. In gel shift assays, the binding we saw with the zygotic *cyclin E* TBE appears to be weaker and less discrete compared to the consensus TBE sequence. The evolutionary goal perhaps at play in MP2 is not to repress *cyclin E* entirely but do it just enough so that the level does not go up excessively. This may be for a reason as evidenced by the finding that when the level of Cyclin E is up-regulated, either in *mid* mutants (Figs 10 and 11) or when *cyclin E* is over-expressed (Fig 13), the lineage generates extra neurons. We did not use *cyclin E* mutants in our studies since a loss of function for *cyclin E* appears to alter the identity of NBs [42]. In the 1960s, Hayflick found that mammalian somatic cells divide 40–60 times and then undergo apoptosis. This is called the Hayflick limit [43]. Precursor cells that give rise to the nervous system do not seem to obey the Hayflick limit. Thus, in Drosophila, for example, neuronal precursors can divide as many as 1200. The extent of cell proliferation may be even greater during the development of our own brain. A Mid-Cyclin E like mechanism such as the one we have shown here might allow these neural cells to escape Hayflick’s limit.

## Materials and methods

### Fly stocks, genetics

*mid* mutant alleles used were *mid*^*1*^, *mid*^*2*^, *los*^*1*^ and a deficiency that removes both *mid* and its sister gene, *H15* (*mid H15*^*df*^ or *mid*^*df*^; Bl# 7498; breakpoints: 25D5-25E6). The other lines used were: *ac-GAL* (BL#: 8715), *UAS-mCD8-GFP* (mouse CD8-GFP transgene under UAS; BL# 41803), *UAS-cyclin E* (BL# 4781), *Hs-cyclin E* (BL# 59056), *UAS-mid* [20], *AJ96-LacZ* [7] RN2 (eve)-GAL4 [21], UAS-tau-LacZ and UAS-tau-GFP [21]. Additional lines used: ftz-GAL4, wg-GAL4 (Kyoto#4918), arm-GAL4 (BM#1561), elav-GAL4 (BM# 8765 or BM#8760), sim-GAL4 (BL#9150). For wild type, we used Oregon R flies. Mutant lines were balanced using GFP-bearing balancer chromosomes or LacZ-bearing balancers to facilitate identification of the mutant genotype. Standard genetics were used to obtain combinations of genotypes when needed.

### Immunohistochemistry

The embryo collection, fixation and immunostaining were performed according to the standard procedures. Briefly, for immunolabeling, embryos were washed thoroughly with running water, dechorionated with 50% bleach, rinsed with running water and then with phosphate-buffered saline containing Triton X-100 (Sigma) (0.1%), and fixed with n-heptane (Fisher Scientific) and 37% formaldehyde (Fisher Scientific) mixed in a 1:1 ratio for 2 min (immunofluorescence labeling) or for 6 min (immunohistochemical labeling). Vitelline membranes were removed by a rapid (~20 seconds) wash with methanol (Fisher Scientific). Embryos were processed immediately. The following antibodies were used: anti-Mid (rabbit, 1:50; ref. 20), anti- Fas II (mouse, 1: 5; DHSB), 22C10 (mouse, 1:1; DHSB), anti-Achaete (mouse, 1:4; source: DHSB), anti-GFP (mouse, 1:100, Abcam, mAb9F9.F9), anti-Odd (guinea pig, 1:100, John Reinitz), anti-Cyclin E (1:4; source: Helena Richardson), anti-β-galactosidase (mouse, 1:500; Cappel), Eve (1:2000; source: Manfred Frasch) and Numb (rabbit, 1:100; Source: Jim Skeath, Chris Doe), Numb (guinea pig, 1:50; source: Jim Skeath), anti-β-galactosidase (rabbit; 1:3000, Invitrogen, A-11132, or mouse, 1:400; DSHB, 40-1a). For color visualization, either AP-conjugated or HRP-conjugated secondary antibodies were used. For light microscopy, secondary antibodies conjugated to alkaline phosphatase (rabbit, 1:200, Pierce, 31341) or horseradish peroxidase (HRP; rabbit, 1:200, Pierce, 31460) were used. Alkaline phosphatase was detected using 5-bromo-4- chloro-3-indolyl-phosphate and nitro blue tetrazolium (Promega, S3771). HRP was detected with diaminobenzidine (Sigma, D4418). For confocal visualization, secondary antibodies conjugated to Cy5 (rabbit, 1:400, Invitrogen, A10523), fluorescein isothiocyanate (mouse, 1:50, Invitrogen, 62–6511), Alexa Fluor 488 (rabbit or mouse, 1:300, Invitrogen, A-11008 or A-11001), or Alexa Fluor 647 (rabbit or mouse, 1:300, Invitrogen, A-21245 or A-21236) or Alexa Flour 488 (1:400) were used.

### *Hs-cyc E* experiments

AJ96-lac Z transgenic line was introduced to *Hs-cyc E* transgenic background and these *Hs-cyc E; AJ96* embryos were collected for 2 hours at room temperature, allowed to develop for 5 hours at room temperature (5–7 hpf embryos). These embryos were then shifted to 30 ^0^C for 30 min, and further allowed to develop for 3–4 hours before fixing and staining for Odd or AJ96 and Odd. The cells were visualized using confocal microscopy.

### Gel shift assay

The consensus TBE and the TBE from the *cyclin E* zygotic promoter were purchased as double-strand oligos from IDT (Integrated DNA Technologies, San Diego, CA). The double-stranded oligos were end-labeled with [^32^P]-ATP with T4 polynucleotide kinase and incubated the Mid protein tagged with 6x Histidine (His) at the C-terminus (MidHis) at different concentrations (0.04 μg, 0.2 μg and 0.4 μg). The *mid-his* fusion gene was expressed in *E*. *coli* BL21-CodonPlus (Stratagene) and the native protein was affinity purified using Ni-NTA resin columns (QIAGEN standard protocol). About 30 pg of the labeled probe and various amounts of the MidHs protein were allowed to interact for 30 minutes on ice in binding buffer (10 mM Tris–HCl, pH 7.5, 50 mM NaCl, 1 mM MgCl2, 0.5 mM DIT, 50 μg/ml poly dl-dC and 4% glycerol) in a final volume of 30μl. The products of the binding reaction were separated using non-denaturing acrylamide: bis-acrylamide gel (4:0.05%) in low ionic strength buffer (6.7 mM Tris, pH 7.5; 1 mM EDTA, pH 7.5, and 3.5 mM sodium acetate) at 4°C. The gel was dried and visualized by autoradiography.

### ImageJ analysis

We used plot profile function to determine the level-distribution profile of Mid and AJ96 (Fig 8) or Cyclin E and Odd (see Figs 10 and 11). The following steps were adopted: for Fig 8, images were saved as 200-pixels/Inch resolution in Adobe Photoshop, and then converted into JPEG files. The JPEG images were opened with ImageJ with the following measurement setup: Area, mean gray value and integrated density were set under Measurements. Using the rectangle function, the area in the cell was defined and under Analyze, the Plot profile function was used for analysis. Since the expression of Mid in one group of MP2 was baseline, we did not convert the gray values into numbers, therefore no statistics were applied (a 0 versus >0 situation). For the analysis of Cyclin E and Odd in MP2 and dMP2 between control versus *mid* at different developmental time points (Figs 10 and 11), ImageJ analysis was done using the plot profile function. The images were set at resolution 300-Pixels/Inch and 3660 Pixels, saved as JPEG files for ImageJ analysis. See Supplementary Information for the dataset and statistical analysis (S4 Data; S1 Statistics).

### Statistics

We used Two Sample T-Test (Welch’s T-Test) to analyze the means between groups of datasets (http://www.statskingdom.com/150MeanT2uneq.html). The details and the analyses are given Supplementary Information (S5 Data).

## Supporting information

S1 Data Supporting information for Figs 1–4 and Table 1. Wild-type and *mid*^*df*^ embryos were stained with different antibodies as shown in Figs 1–4 and the various MP2 lineage defects were counted and recorded. The results were tabulated in Table 1.
(DOCX)

S2 Data Supporting information for Fig 7. Wild-type and *mid* mutant embryos from 5 hpf and 6 hpf old embryos were stained with Ac and Numb and analyzed by confocal microscopy. The penetrance of the aberrant and normal Numb localization in MP2 was recorded.
(DOCX)

S3 Data Supporting information for Fig 9. Effect of over-expression of Mid in MP2: The gain of function effects was examined by staining embryos with Ac, AJ, or Odd and the penetrance of the phenotypes was recorded.
(DOCX)

S4 Data Supporting information for Figs 10 and 11. The intensity of expression of Cyclin E and Odd was measured in MP2 (5–5.5 hpf and 5.5–6.0 hpf) and dMP2 (8–8.5 hpf) using the ImageJ software by measuring the plot profile across MP2/dMP2 cells. The statistical analysis of the means between groups of datasets was done using the Two-Sample T-Test (Welch’s T-Test).
(DOCX)

S5 Data Supporting information for Fig 13. *UAS-cyclin E* and heat shock70 promoter driven *cyclin E* (*Hs-cyclin E*) were induced either with ac-GAL4 or a brief heat shock treatment (see Materials and methods) and the embryos were stained for Odd or Odd and AJ96 expression. The MP2 phenotypes were recorded in each genotype.
(DOCX)

S1 Statistics Statistical analysis of the datasets from Figs 10 and 11. Two Sample T-Test (Welch’s T-Test) analysis of the means between groups of datasets. **Statistics:** Wild-type control vs *mid* mutant: Cyclin E (5–5.5 hr) in MP2: wild-type versus *mid*: P = 0.631777 (H0 is accepted) Odd (5–5.5 hr) in MP2: wild-type versus *mid*: P = 0.0146960 (H0 is rejected) Cyclin E (5.5–6.0) in MP2: wild-type versus *mid*: P = 5.61231e-7(H0 is rejected) Odd (5.5–6.0) in MP2: wild-type versus *mid*: P = 0.285161 (H0 is accepted) Cyclin E (8–8.5) in dMP2: wild-type versus *mid*: P = 0.679337(H0 is accepted) Odd (8–8.5 hr) in dMP2: wild-type versus *mid*: P = 0. 00496460(H0 is rejected) Between 5.0–5.5 hpf vs 5.5–6.0, control and *mid*: Control—Cyclin E: P = 0.000155669 (H0 is rejected); Odd: P = 0.0865129 (Ho is accepted) *mid*—Cyclin E: P = 0.0000448175 (H0 is rejected); Odd: P = 0.00152362 (H0 is rejected) Between 5.5–6.0 hpf vs 8–8.5, control and *mid*: Control—Cyclin E: P = 0.432896 (H0 is accepted); Odd: P = 0.000408976 (Ho is rejected) *mid*—Cyclin E: P = 2.58473e-7 (H0 is rejected); Odd: P = 0.833928 (H0 is accepted) Between 5–5.5 hpf vs 8–8.5, control and *mid*: Control—Cyclin E: P = 0.0000181578 (H0 is accepted); Odd: P = 0.00105391(Ho is rejected) *mid*—Cyclin E: P = 0.0000790914 (H0 is rejected); Odd: P = 0.00673910(H0 is rejected).
(DOCX)
