# Supplementary material for: Restriction on self-renewing asymmetric division is coupled to terminal asymmetric division in the Drosophila CNS
Source: PLoS Genet. 2020 Sep 28;16(9):e1009011. doi: 10.1371/journal.pgen.1009011 (PMC7521697; doi:10.1371/journal.pgen.1009011)
Supplement: S1 Data — Wild-type and middf embryos were stained with different antibodies as shown in Figs 1–4 and the various MP2 lineage defects were counted and recorded. The results were tabulated in Table 1. (DOCX) [file pgen.1009011.s001.docx]

**
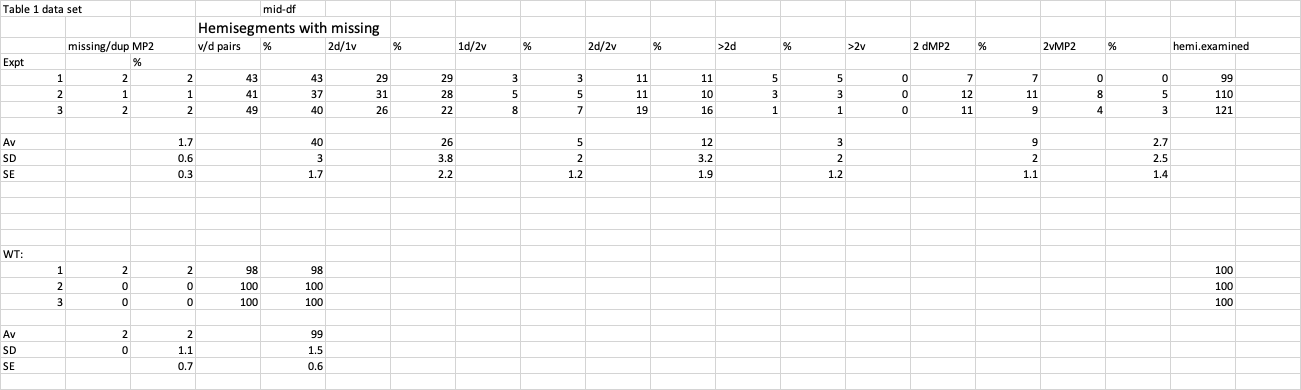
**

**Supporting Information for Figs 1-4 and Table 1**: Wild-type and *mid^df^* embryos were stained with different

antibodies as shown in Figs 1-4 and the various MP2 lineage defects were counted and recorded. The results were tabulated in Table 1.
