# Supplementary material for: Restriction on self-renewing asymmetric division is coupled to terminal asymmetric division in the Drosophila CNS
Source: PLoS Genet. 2020 Sep 28;16(9):e1009011. doi: 10.1371/journal.pgen.1009011 (PMC7521697; doi:10.1371/journal.pgen.1009011)
Supplement: S2 Data — Wild-type and mid mutant embryos from 5 hpf and 6 hpf old embryos were stained with Ac and Numb and analyzed by confocal microscopy. The penetrance of the aberrant and normal Numb localization in MP2 was recorded. (DOCX) [file pgen.1009011.s002.docx]

| Fig. 7 |  |  |  |  |  |  |  |  |
| --- | --- | --- | --- | --- | --- | --- | --- | --- |
|  |  |  |  |  |  |  |  |  |
| Ac+Numb |  |  |  | WT |  |  |  |  |
|  |  | Expt | Asymmetric Numb | | non-Asy.Numb | | # of hemi.counted | |
|  |  | 1 | 36 | 95.00% | 2 | 5.00% | 38 |  |
| 5-5 hrs old embryos | | 2 | 44 | 92.00% | 4 | 8.00% | 48 |  |
|  |  | 3 | 33 | 87.00% | 5 | 13.00% | 38 |  |
|  |  |  | Av | 91.00% |  | 8.60% |  |  |
|  |  |  | SD | 4.00% |  | 4.00% |  |  |
|  |  |  | SE | 2.30% |  | 2.30% |  |  |
|  |  |  |  |  |  |  |  |  |
|  |  |  |  |  |  |  |  |  |
|  |  |  |  | mid |  |  |  |  |
|  |  | Expt | Asymmetric Numb | | Non-Asymmetric Numb | | # of hemi counted | |
|  |  | 1 | 15 | 68% | 7 | 32% | 22 |  |
|  |  | 2 | 14 | 58% | 10 | 42% | 24 |  |
|  |  | 3 | 18 | 60% | 12 | 40% | 30 |  |
|  |  |  | Av | 62% |  | 38% |  |  |
|  |  |  | SD | 5% |  | 5% |  |  |
|  |  |  | SE | 3% |  | 3% |  |  |
|  |  |  |  |  |  |  |  |  |
|  |  |  |  |  |  |  |  |  |
|  |  |  |  | WT |  |  |  |  |
|  |  | Expt | Asymmetric Numb | | Non-Asymmetric Numb | | # of hemi counted | |
|  |  | 1 | 28 | 93% | 2 | 7% | 30 |  |
|  |  | 2 | 26 | 96% | 1 | 4% | 27 |  |
| 6 hrs old embryos | | 3 | 25 | 100% | 0 | 0% | 25 |  |
|  |  |  | Av | 96% |  | 4% |  |  |
|  |  |  | SD | 4% |  | 4% |  |  |
|  |  |  | SE | 2% |  | 2% |  |  |
|  |  |  |  |  |  |  |  |  |
|  |  |  |  |  |  |  |  |  |
|  |  |  |  | mid |  |  |  |  |
|  |  | Expt | Asymmetric Numb | | Non-Asymmetric Numb | | # of hemi counted | |
|  |  | 1 | 15 | 71% | 6 | 29% | 21 |  |
|  |  | 2 | 13 | 72% | 5 | 28% | 18 |  |
|  |  | 3 | 11 | 61% | 7 | 39% | 18 |  |
|  |  |  | Av | 68% |  | 32% |  |  |
|  |  |  | SD | 6% |  | 6% |  |  |
|  |  |  | SE | 4% |  | 4% |  |  |

**Supporting Information for Fig 7**: Wild-type and *mid* mutant embryos from 5 hpf and 6 hpf old embryos were stained with Ac and Numb and analyzed by confocal microscopy. The penetrance of the aberrant and normal Numb localization in MP2 was recorded.
