# Supplementary material for: Restriction on self-renewing asymmetric division is coupled to terminal asymmetric division in the Drosophila CNS
Source: PLoS Genet. 2020 Sep 28;16(9):e1009011. doi: 10.1371/journal.pgen.1009011 (PMC7521697; doi:10.1371/journal.pgen.1009011)
Supplement: S3 Data — Effect of over-expression of Mid in MP2: The gain of function effects was examined by staining embryos with Ac, AJ, or Odd and the penetrance of the phenotypes was recorded. (DOCX) [file pgen.1009011.s003.docx]

**
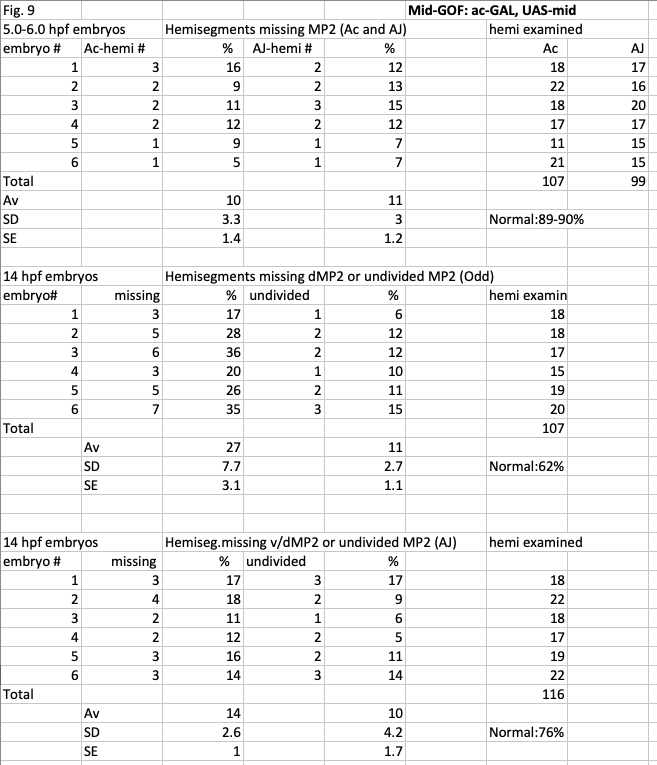
Supporting Information for Fig 9**: Effect of over-expression of Mid in MP2: The gain of function effects was examined by staining embryos with Ac, AJ, or Odd and the penetrance of the phenotypes was recorded.
