# Supplementary material for: Restriction on self-renewing asymmetric division is coupled to terminal asymmetric division in the Drosophila CNS
Source: PLoS Genet. 2020 Sep 28;16(9):e1009011. doi: 10.1371/journal.pgen.1009011 (PMC7521697; doi:10.1371/journal.pgen.1009011)
Supplement: S4 Data — The intensity of expression of Cyclin E and Odd was measured in MP2 (5–5.5 hpf and 5.5–6.0 hpf) and dMP2 (8–8.5 hpf) using the ImageJ software by measuring the plot profile across MP2/dMP2 cells. The statistical analysis of the means between groups of datasets was done using the Two-Sample T-Test (Welch’s T-Test). (DOCX) [file pgen.1009011.s004.docx]

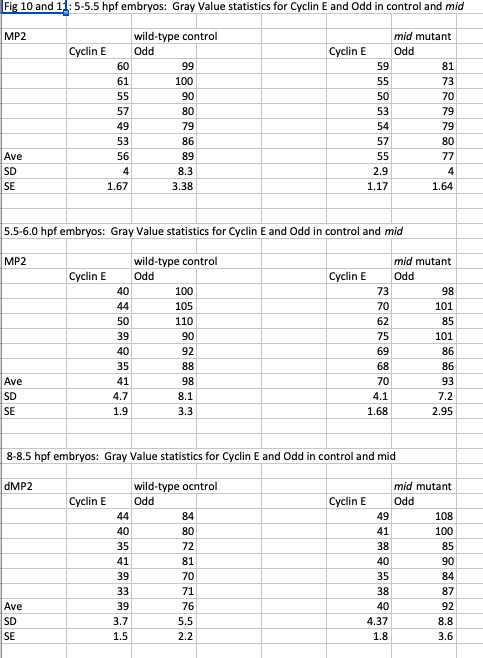


**Supporting Information for Fig 10 and 11.** The intensity of expression of Cyclin E and Odd was measured in MP2 (5-5.5 hpf and 5.5-6.0 hpf) and dMP2 (8-8.5 hpf) using the ImageJ software by measuring the plot profile across MP2/dMP2 cells. The statistical analysis of the means between groups of datasets was done using the Two-Sample T-Test (Welch’s T-Test).

**Statistics:**

Wild-type control vs *mid* mutant:

Cyclin E (5-5.5 hr) in MP2: wild-type versus *mid*: P= 0.631777 (H0 is accepted)

Odd (5-5.5 hr) in MP2: wild-type versus *mid*: P= 0.0146960 (H0 is rejected)

Cyclin E (5.5-6.0) in MP2: wild-type versus *mid*: P= 5.61231e-7(H0 is rejected)

Odd (5.5-6.0) in MP2: wild-type versus *mid*: P= 0.285161 (H0 is accepted)

Cyclin E (8-8.5) in dMP2: wild-type versus *mid*: P= 0.679337(H0 is accepted)

Odd (8-8.5 hr) in dMP2: wild-type versus *mid*: P=0. 00496460(H0 is rejected)

Between 5.0-5.5 hpf vs 5.5-6.0, control and *mid:*

Control-- Cyclin E: P=0.000155669 (H0 is rejected); Odd: P=0.0865129 (Ho is accepted)

*mid*—Cyclin E: P= 0.0000448175 (H0 is rejected); Odd: P=0.00152362 (H0 is rejected)

Between 5.5-6.0 hpf vs 8-8.5, control and *mid*:

Control-- Cyclin E: P=0.432896 (H0 is accepted); Odd: P=0.000408976 (Ho is rejected)

*mid*—Cyclin E: P= 2.58473e-7 (H0 is rejected); Odd: P=0.833928 (H0 is accepted)

Between 5-5.5 hpf vs 8-8.5, control and *mid*:

Control-- Cyclin E: P=0.0000181578 (H0 is accepted); Odd: P=0.00105391(Ho is rejected)

*mid*—Cyclin E: P=0.0000790914 (H0 is rejected); Odd: P= 0.00673910(H0 is rejected)
