# Supplementary material for: Restriction on self-renewing asymmetric division is coupled to terminal asymmetric division in the Drosophila CNS
Source: PLoS Genet. 2020 Sep 28;16(9):e1009011. doi: 10.1371/journal.pgen.1009011 (PMC7521697; doi:10.1371/journal.pgen.1009011)
Supplement: S5 Data — UAS-cyclin E and heat shock70 promoter driven cyclin E (Hs-cyclin E) were induced either with ac-GAL4 or a brief heat shock treatment (see Materials and methods) and the embryos were stained for Odd or Odd and AJ96 expression. The MP2 phenotypes were recorded in each genotype. (DOCX) [file pgen.1009011.s005.docx]

| Fig. 13: |  |  |  |  |  |  |  |  |
| --- | --- | --- | --- | --- | --- | --- | --- | --- |
|  |  | **ac-GAL4/*UAS-cyclin E* dataset** | | | |  |  |  |
|  |  |  |  |  |  |  |  |  |
|  | dMP2/vMP2 |  | 2dMP2/1vMP2 | | 1dMP2/2vMP2 | |  |  |
|  | no. | % | no. | % | no | % | total hemi examined | |
| expt 1 | 73 | 55 | 42 | 32 | ND |  | 132 |  |
| expt 2 | 65 | 53 | 43 | 35 |  |  | 122 |  |
| expt 3 | 55 | 50 | 39 | 35 |  |  | 111 |  |
| Av |  | 53 |  | 34 |  |  |  |  |
| SD |  | 2.5 |  | 1.7 |  |  |  |  |
| SE |  | 1.5 |  | 1 |  |  |  |  |
|  |  |  |  |  |  |  |  |  |
|  |  |  |  |  |  |  |  |  |
|  |  | ***Hs-cyclin E* dataset** | |  |  |  |  |  |
|  |  |  |  |  |  |  |  |  |
|  | dMP2/vMP2 |  | 2DMP2/1vMP2 | | 1dMP2/2vMP2 | |  |  |
|  | no. | % | no. | % | no | % | total hemi examined | |
| expt 1 | 55 | 55 | 37 | 37 | 7 | 7 | 99 |  |
| expt 2 | 51 | 64 | 25 | 31 | 4 | 5 | 80 |  |
| expt 3 | 41 | 63 | 21 | 32 | 3 | 4.6 | 65 |  |
| Av |  | 61 |  | 33 |  | 5.5 |  |  |
| SD |  | 4.9 |  | 3.2 |  | 1.3 |  |  |
| SE |  | 2.8 |  | 1.8 |  | 0.7 |  |  |

**Supporting Information for Fig 13:** *UAS-cyclin E* and *heat shock70* promoter driven *cyclin E* (*Hs-cyclin E*) were induced either with ac-GAL4 or a brief heat shock treatment (see Materials and methods) and the embryos were stained for Odd or Odd and AJ96 expression. The MP2 phenotypes were recorded in each genotype.
