# Supplementary material for: Restriction on self-renewing asymmetric division is coupled to terminal asymmetric division in the Drosophila CNS
Source: PLoS Genet. 2020 Sep 28;16(9):e1009011. doi: 10.1371/journal.pgen.1009011 (PMC7521697; doi:10.1371/journal.pgen.1009011)

**Supporting Information: Statistical analysis of data for Figs 10 and 11**


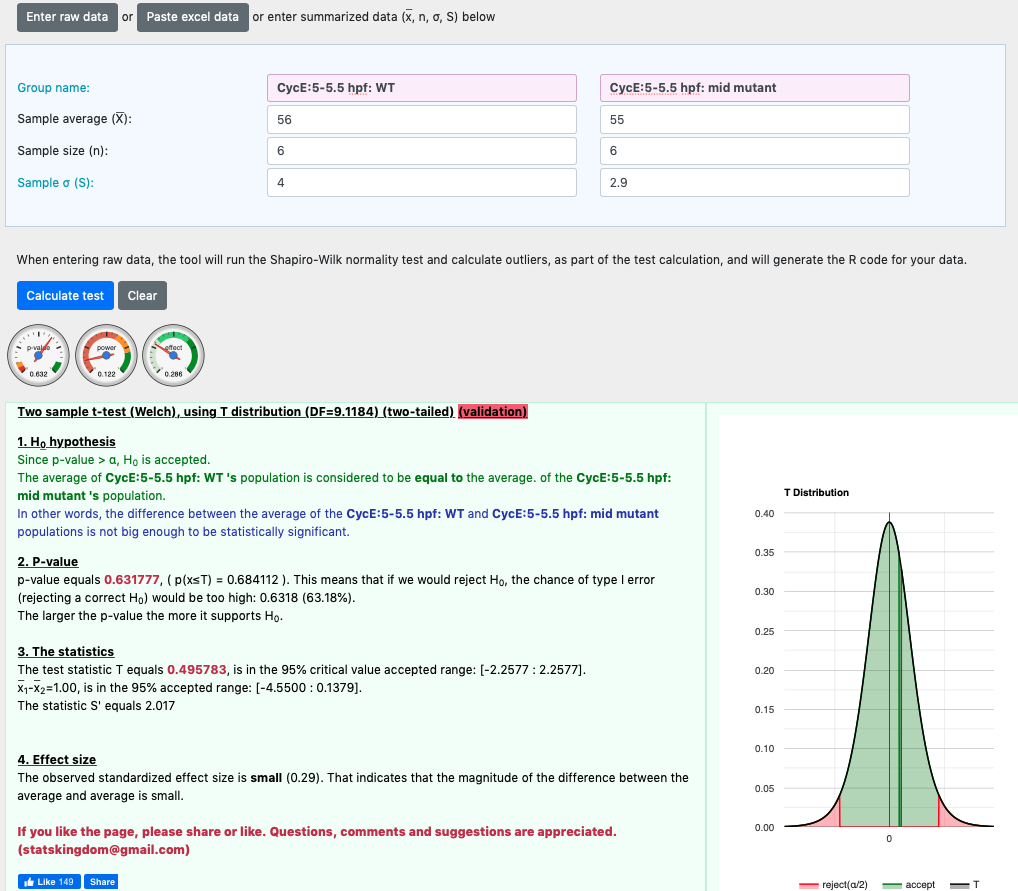
**Cyclin E in MP2 (5-5.5 hpf) in wild-type versus *mid* mutant**

**
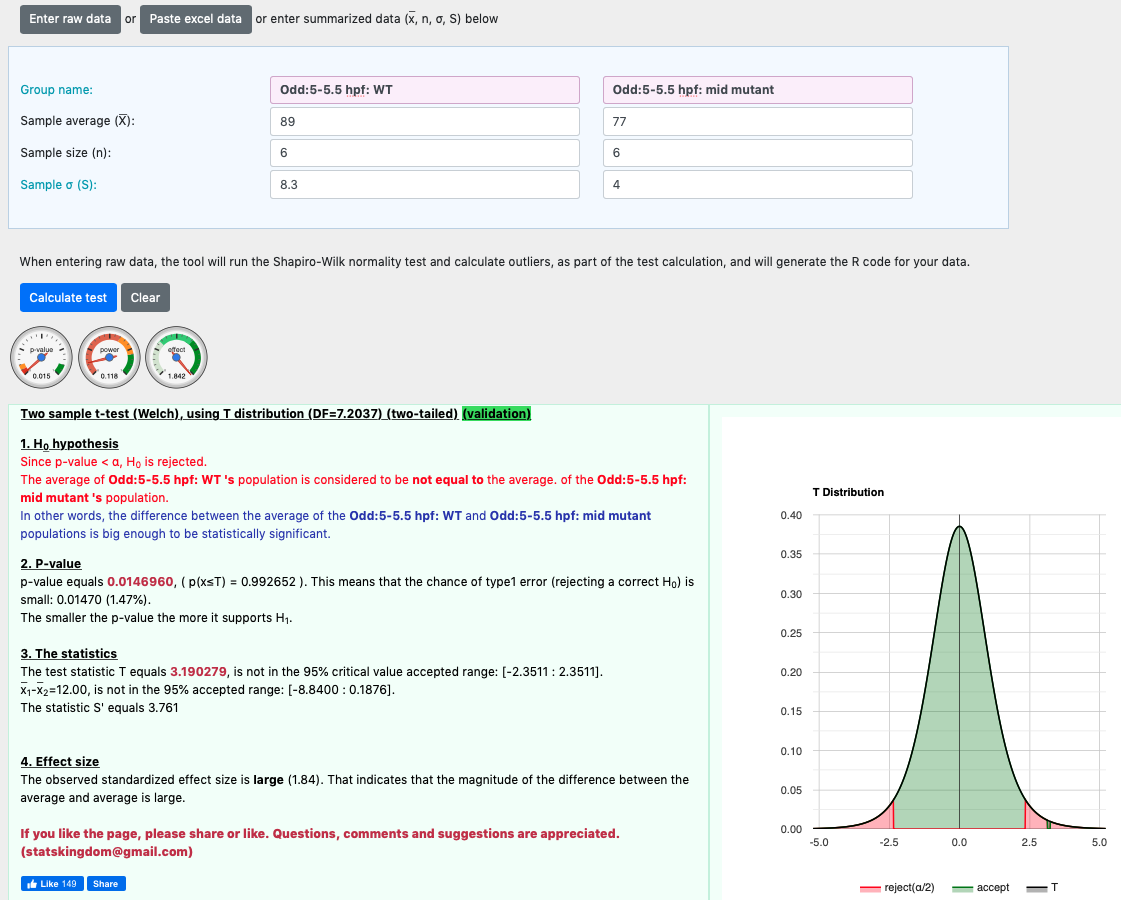
Odd in MP2 (5-5.5 hpf) in wild-type versus *mid* mutant**


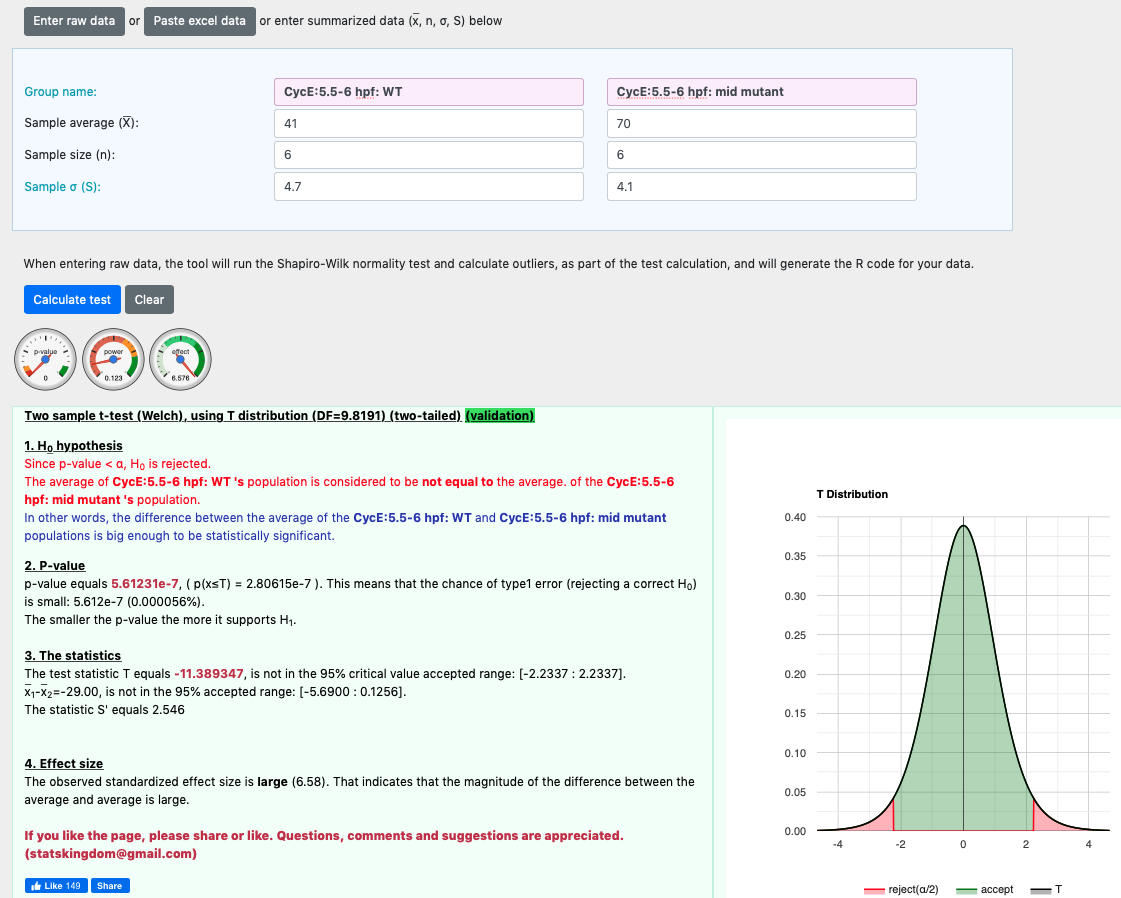
**Cyclin E in MP2 (5.5-6.0 hpf) in wild-type versus *mid* mutant**

**
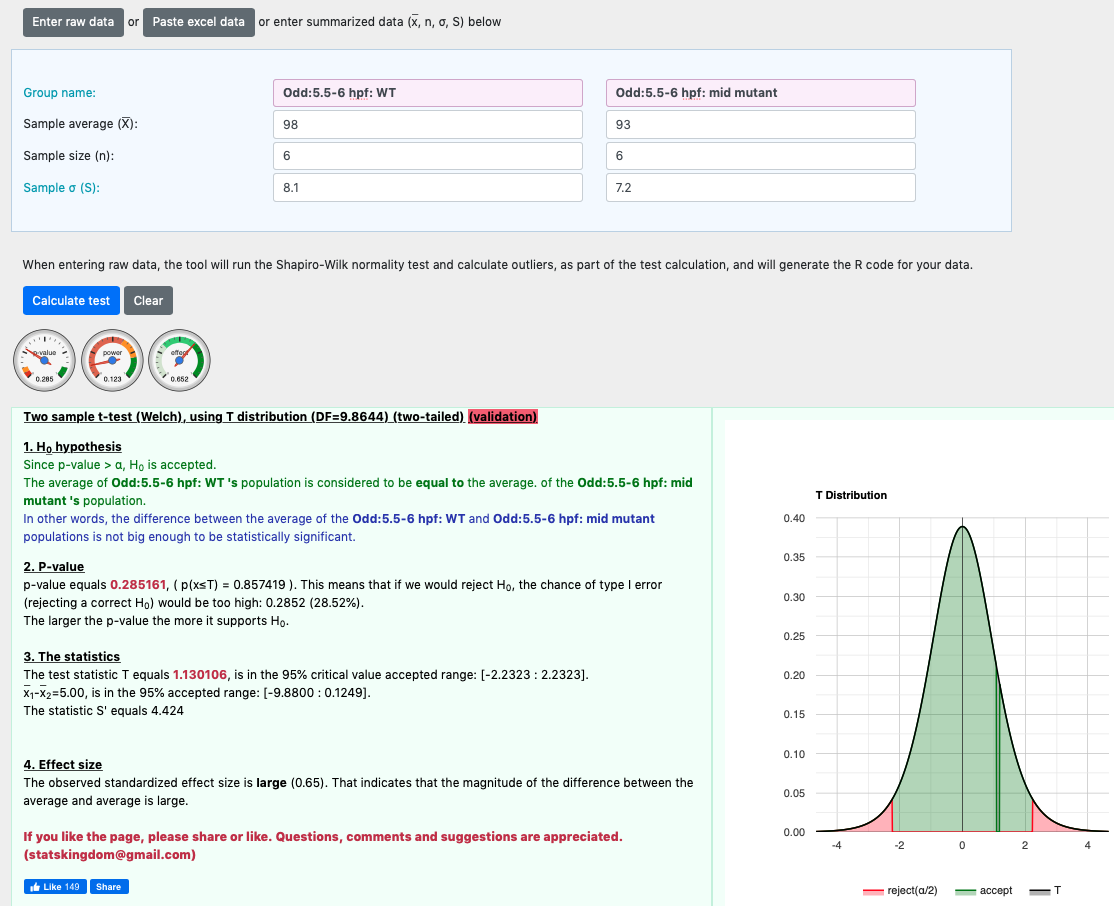
Odd in MP2 (5.5-6.0 hpf) in wild-type versus *mid* mutant**

**Cyclin E in dMP2 (8-8.5 hpf) in wild-type versus *mid* mutant**

**
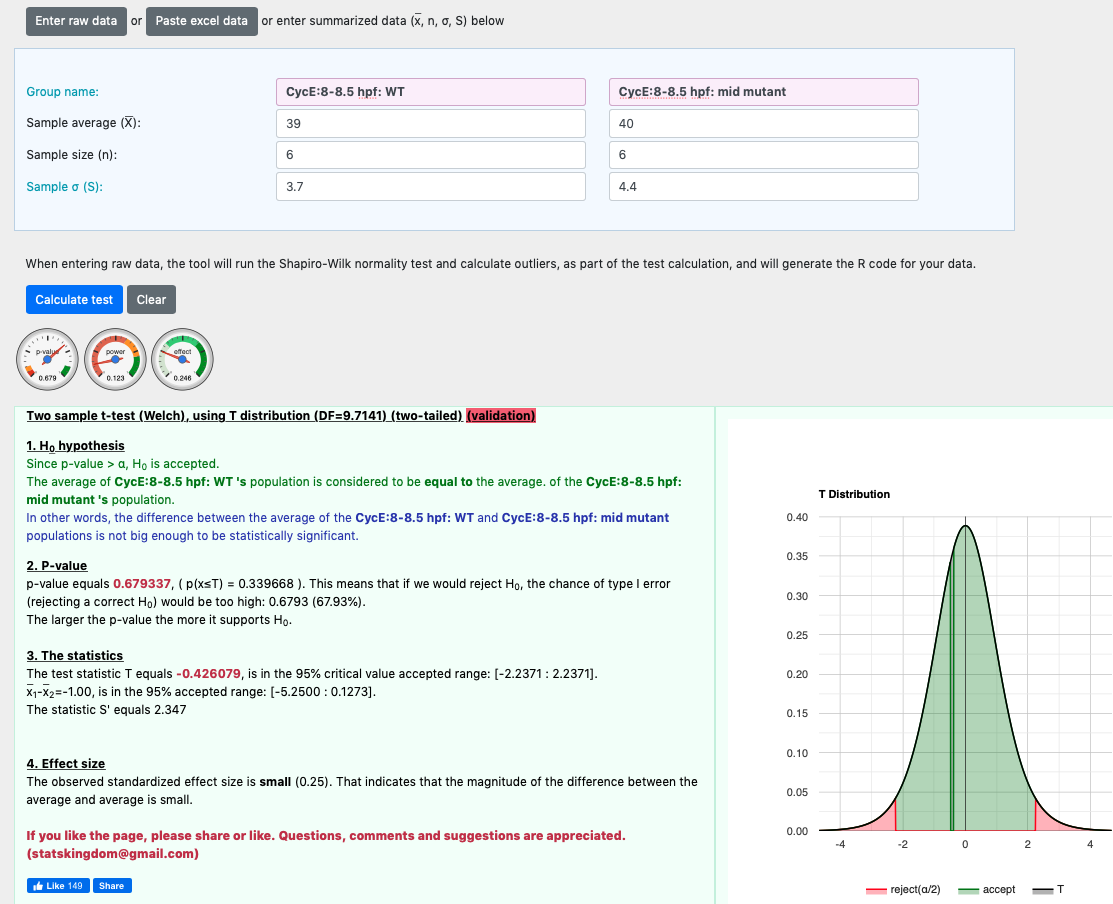
**

**Odd in dMP2 (8-8.5 hpf) in wild-type versus *mid* mutant**

**
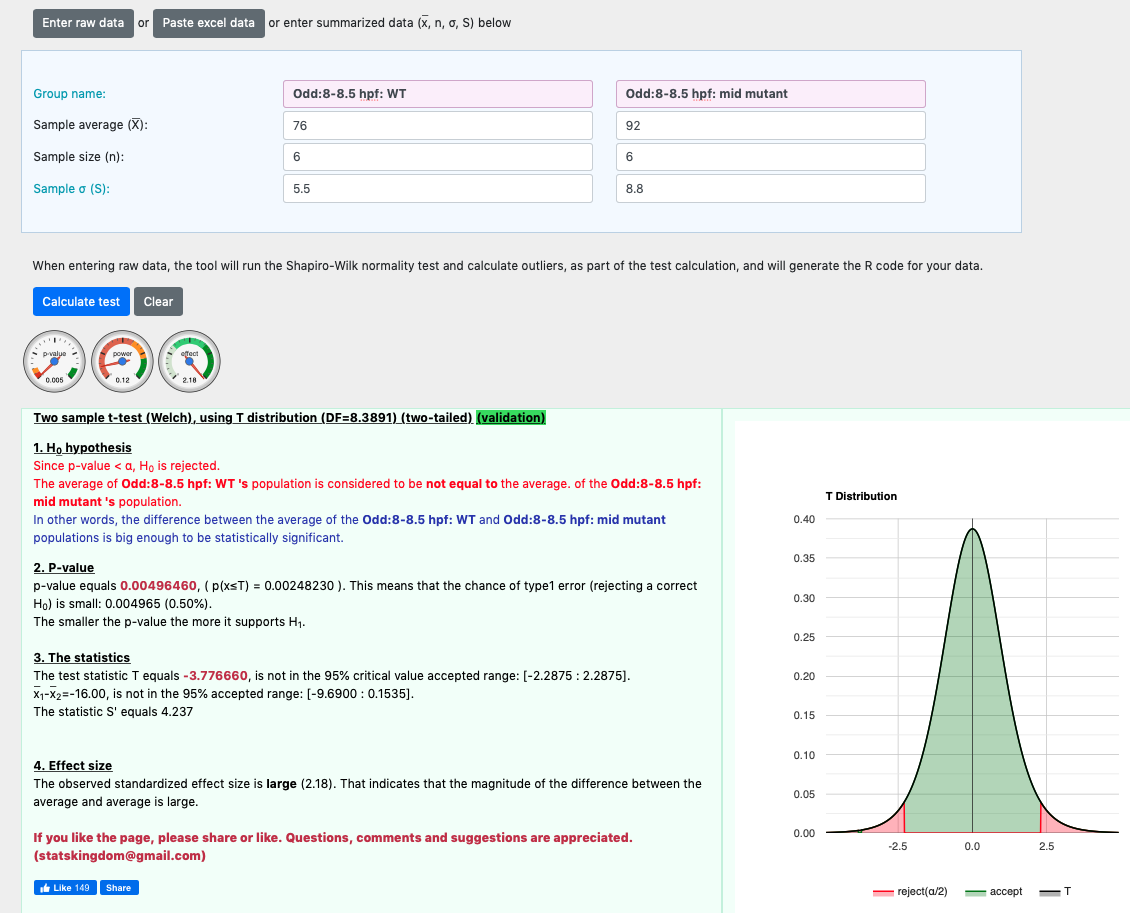
**

**
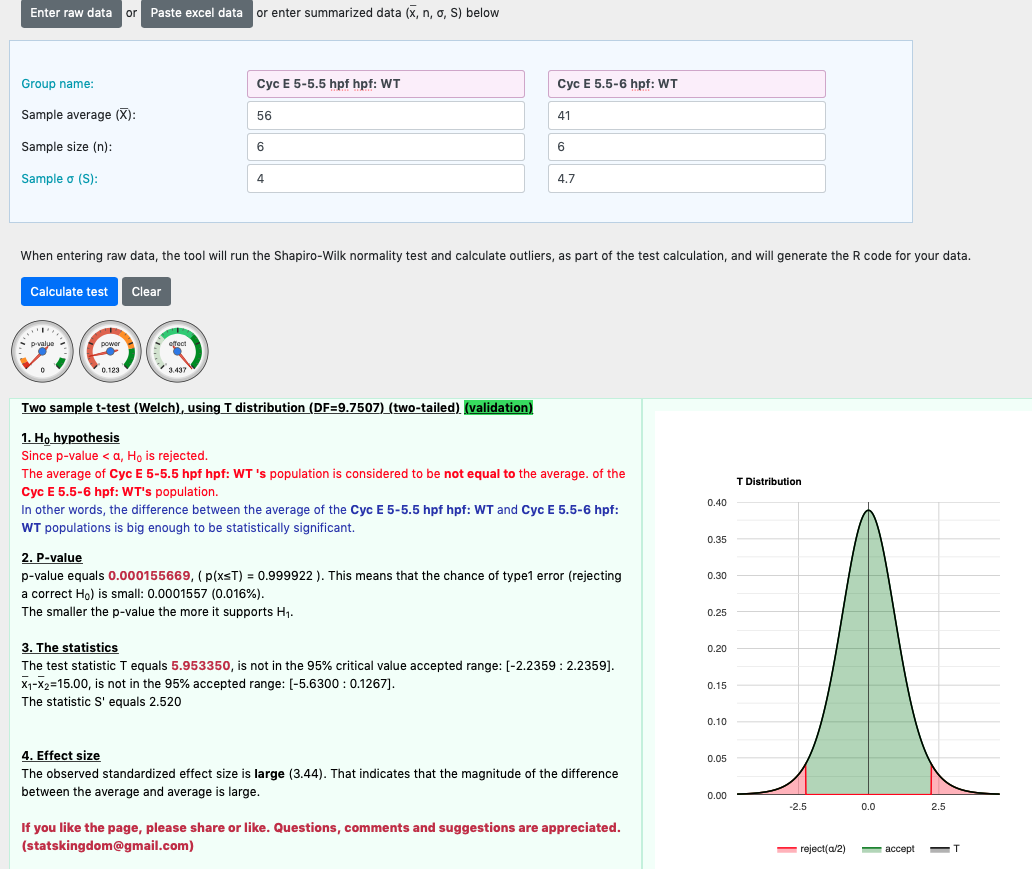
Cyclin E in MP2 (5-5.5 hpf versus 5.5-6 hpf) in wild-type**

**
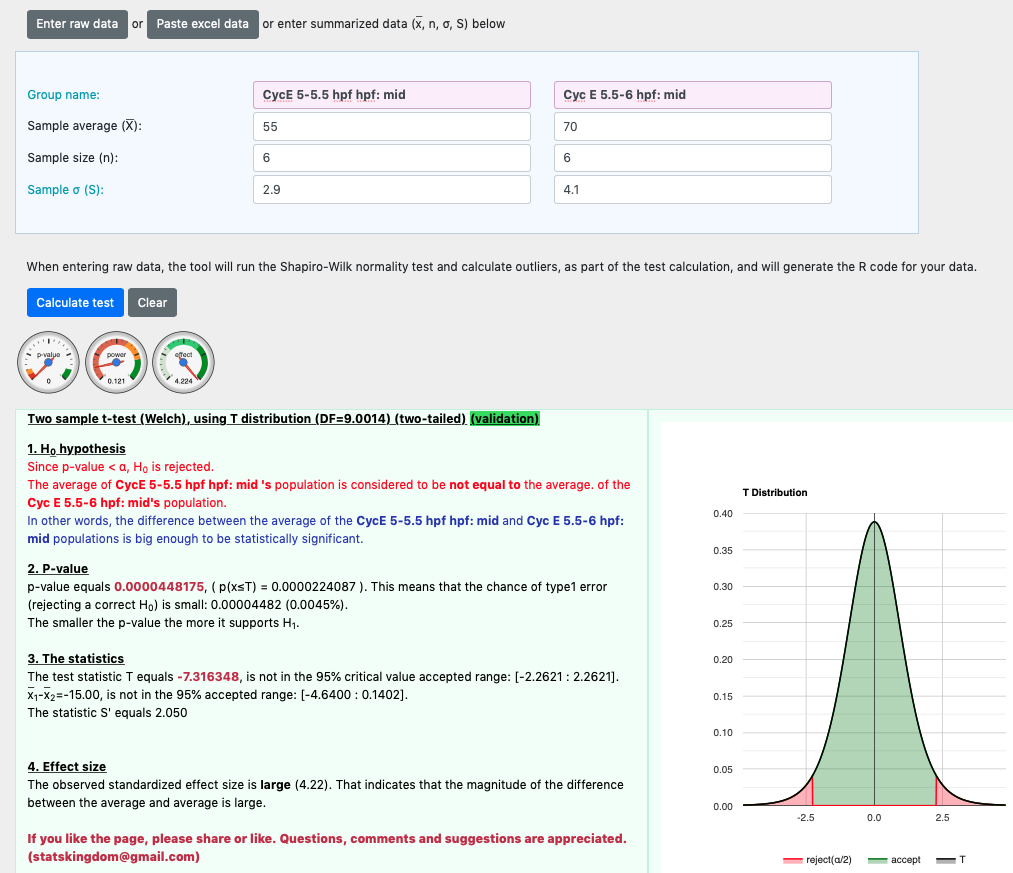
Cyclin E in MP2 (5-5.5 versus 5.5-6 hpf) in *mid***

**
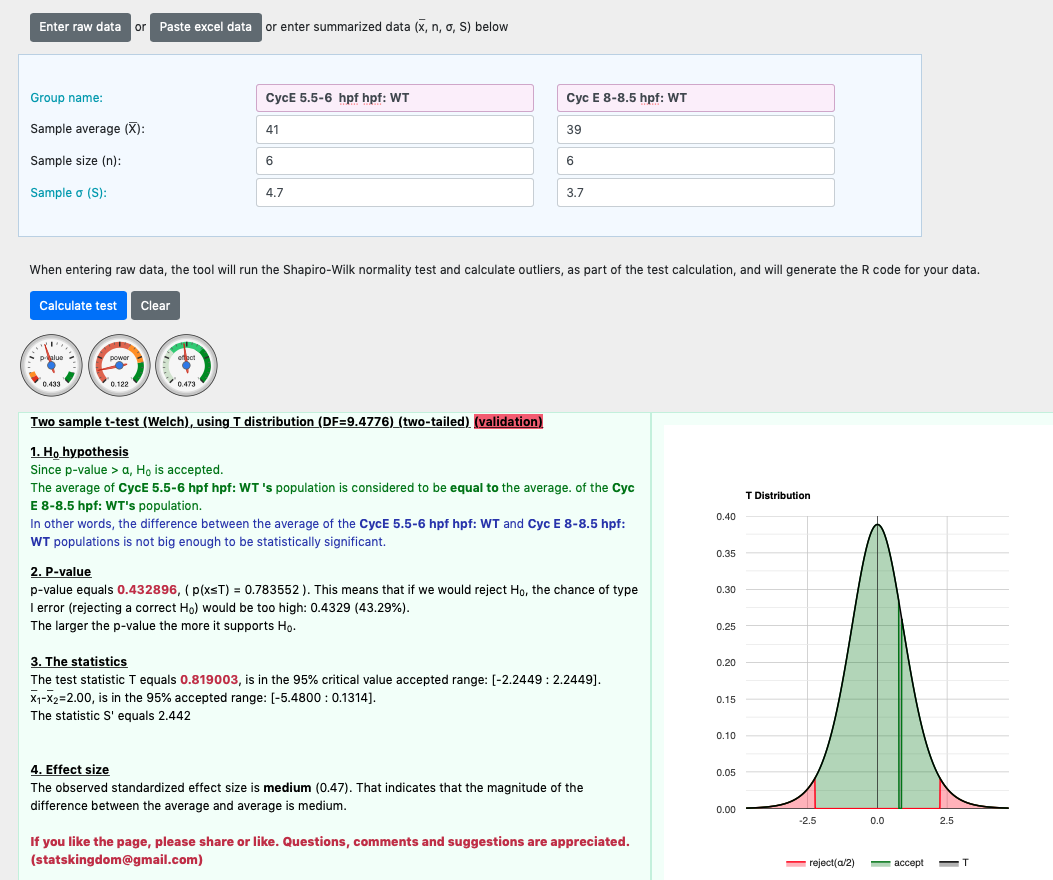
Cyclin E in MP2 (5.5-6 hpf) in wild-type**

**
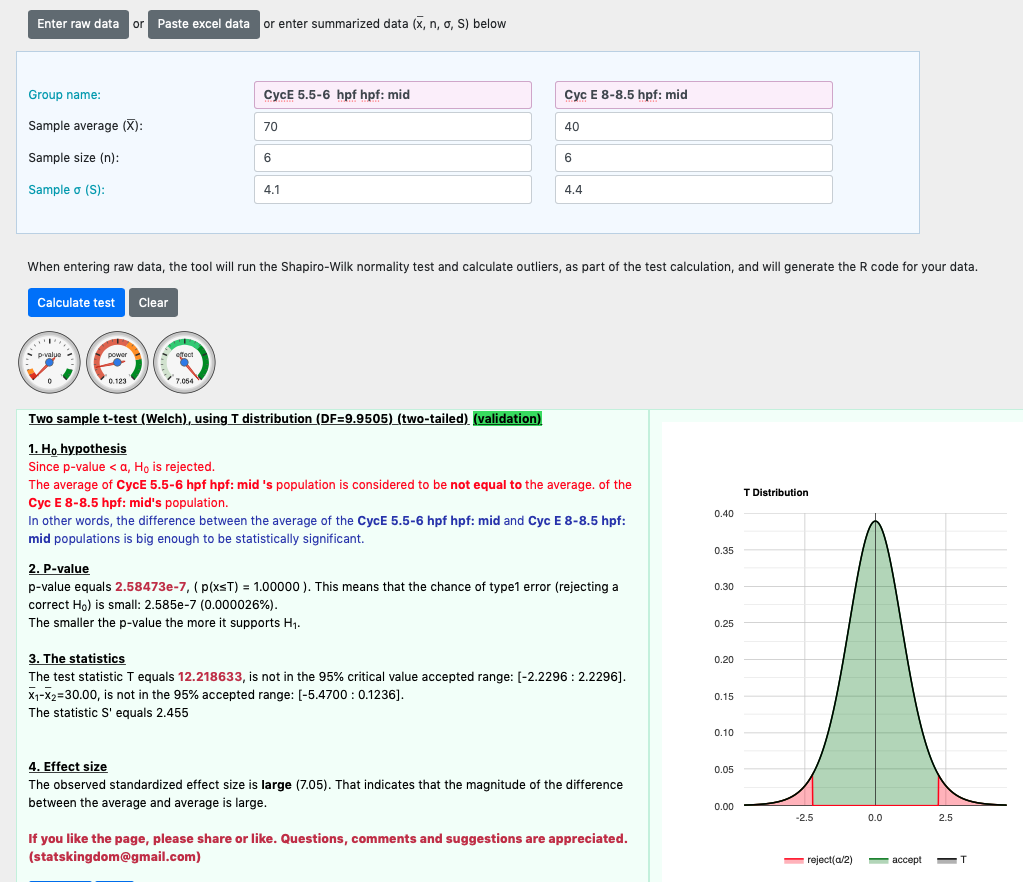
Cyclin E in MP2 (5.5-6 hpf) versus dMP2 (8-8.5 hpf) in *mid***

**
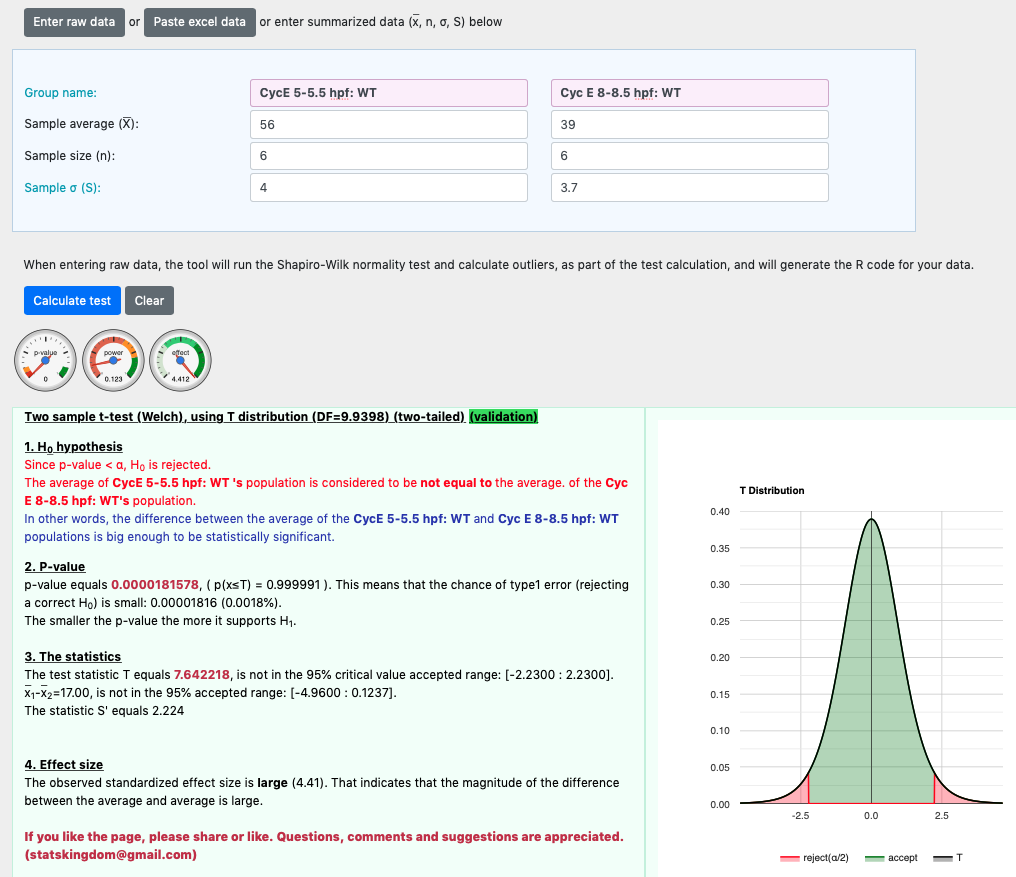
Cyclin E in MP2 (5.5-6 hpf) versus dMP2 (8-8.5 hpf) in wild-type**

**
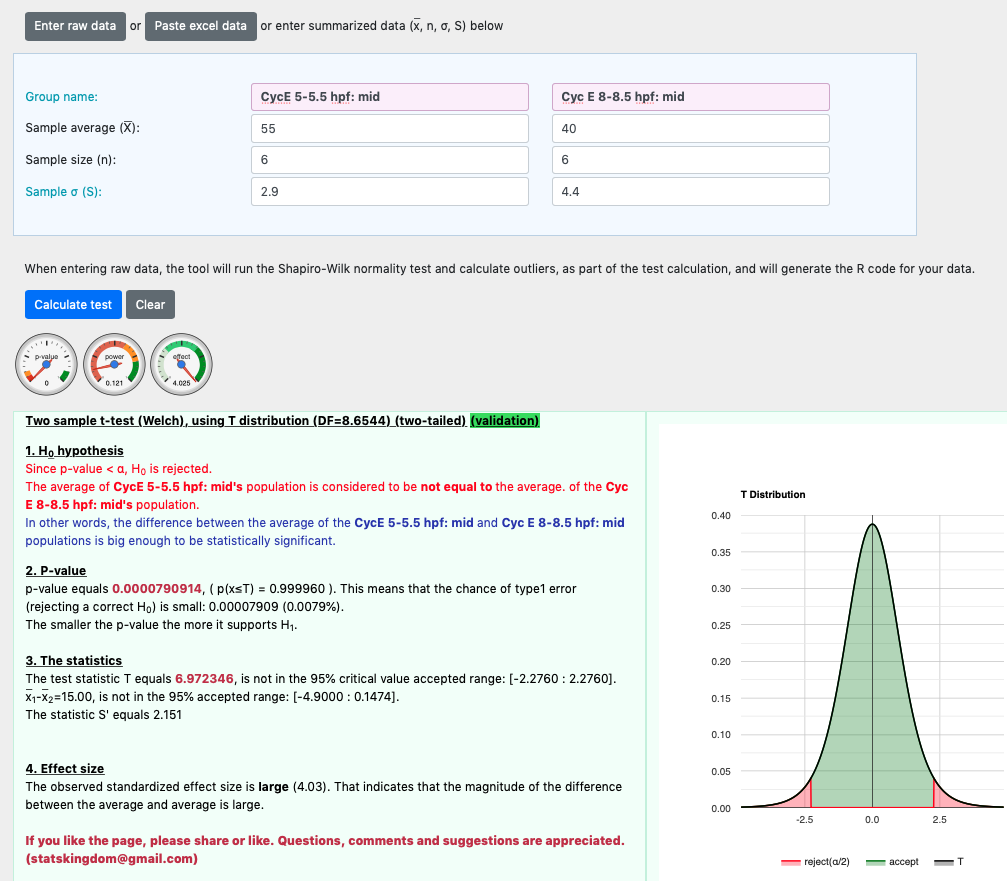
Cyclin E in MP2 (5-5.5 hpf) versus dMP2 (8-8.5 hpf) in *mid***

**
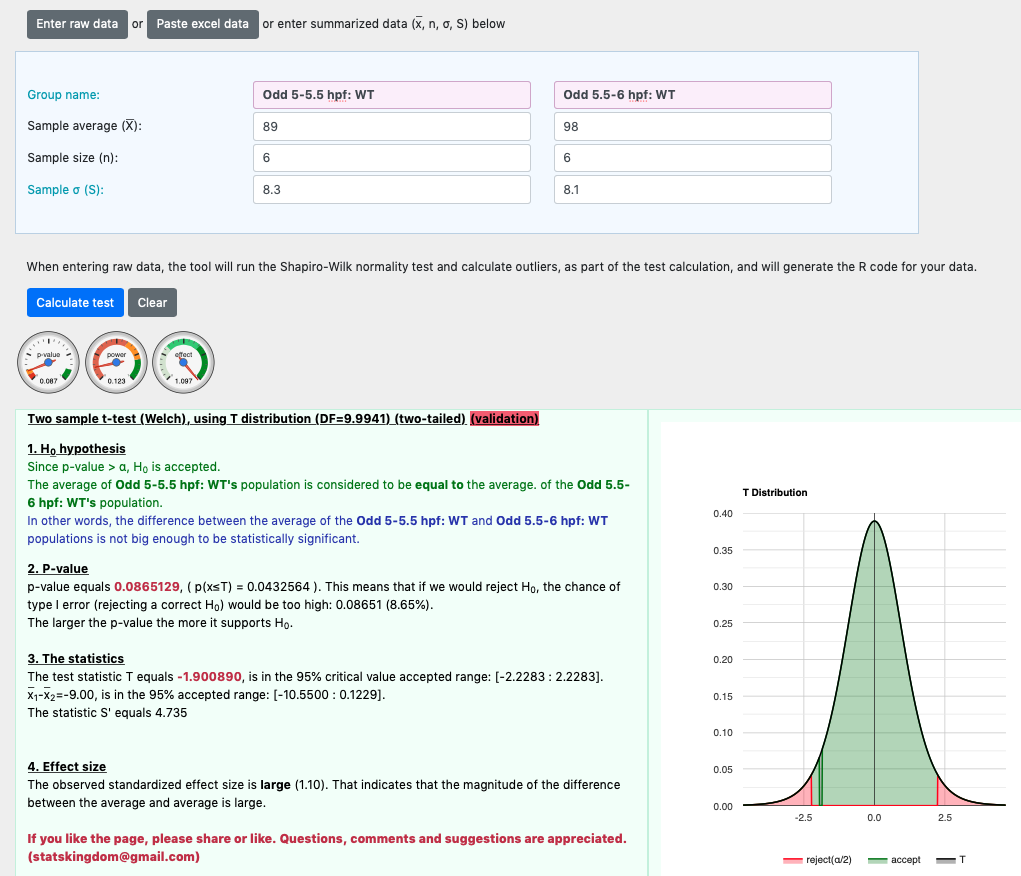
Odd in MP2 (5-5.5 hpf) in wild-type**

**
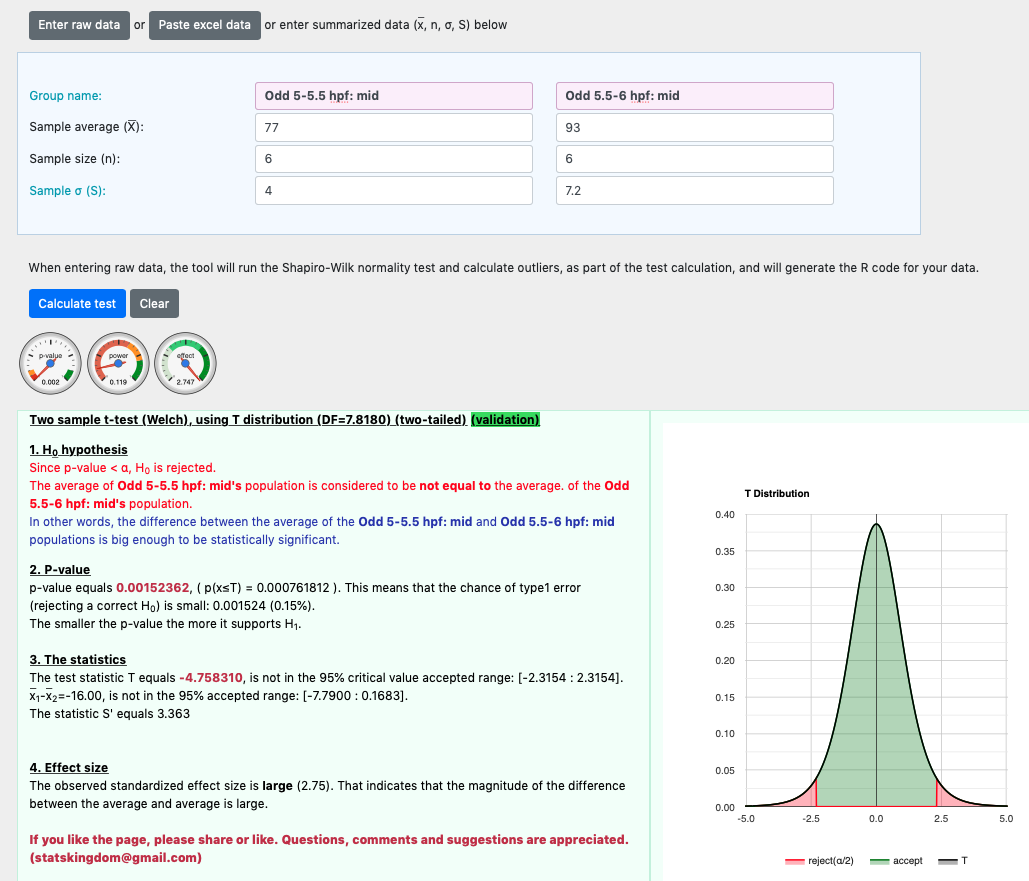
Odd in MP2 (5-5.5 hpf) in *mid***


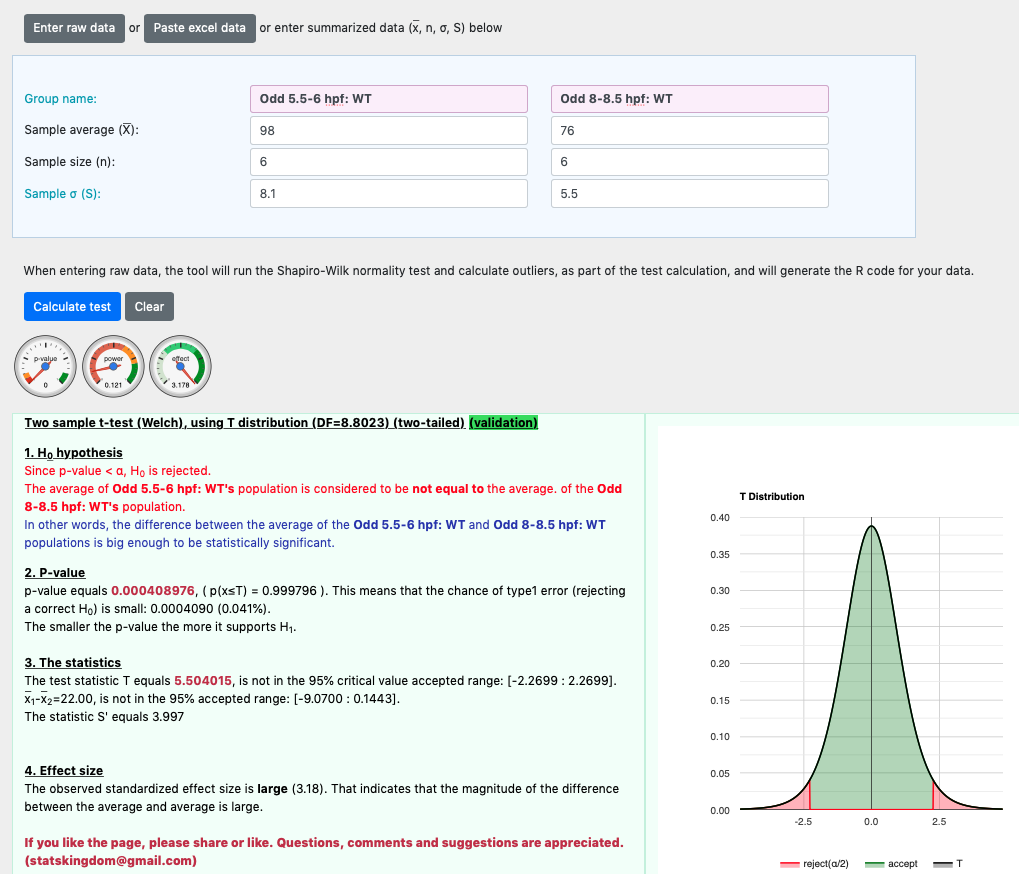
**Odd in MP2 (5.5-6 hpf) versus dMP2 (8-8.5 hpf) in wild-type**

**
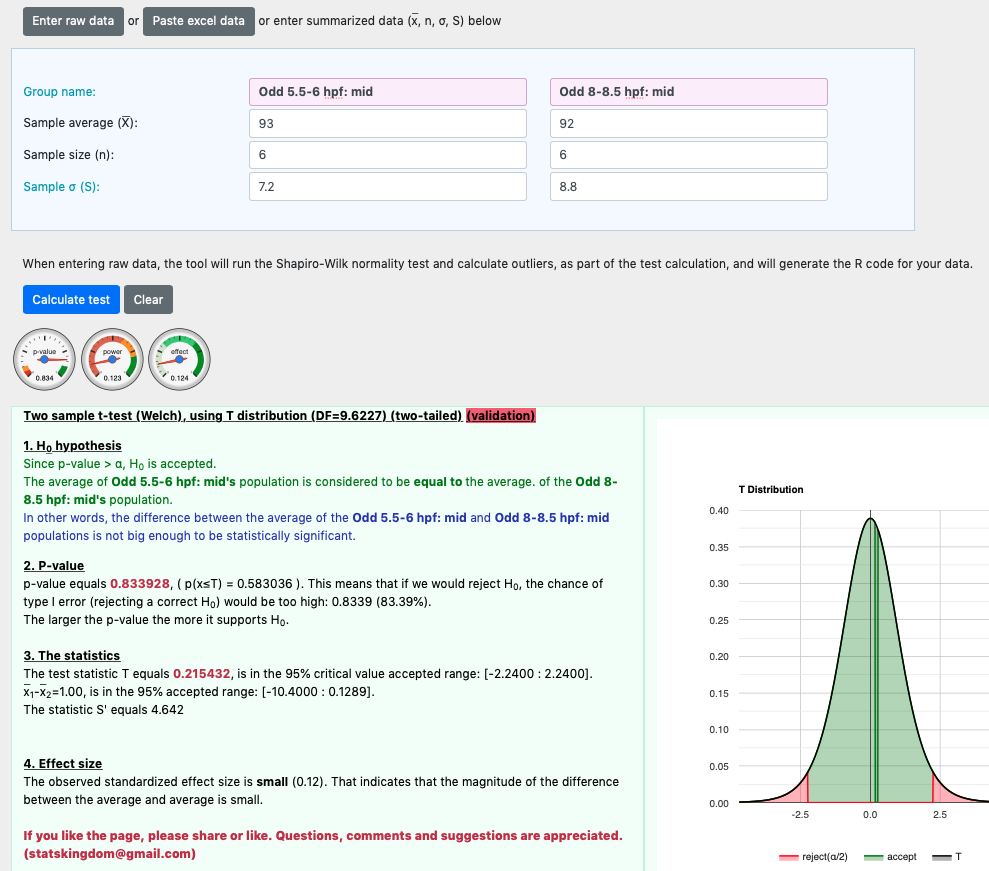
Odd in MP2 (5.5-6 hpf) versus dMP2 (8-8.5 hpf) in *mid***

**
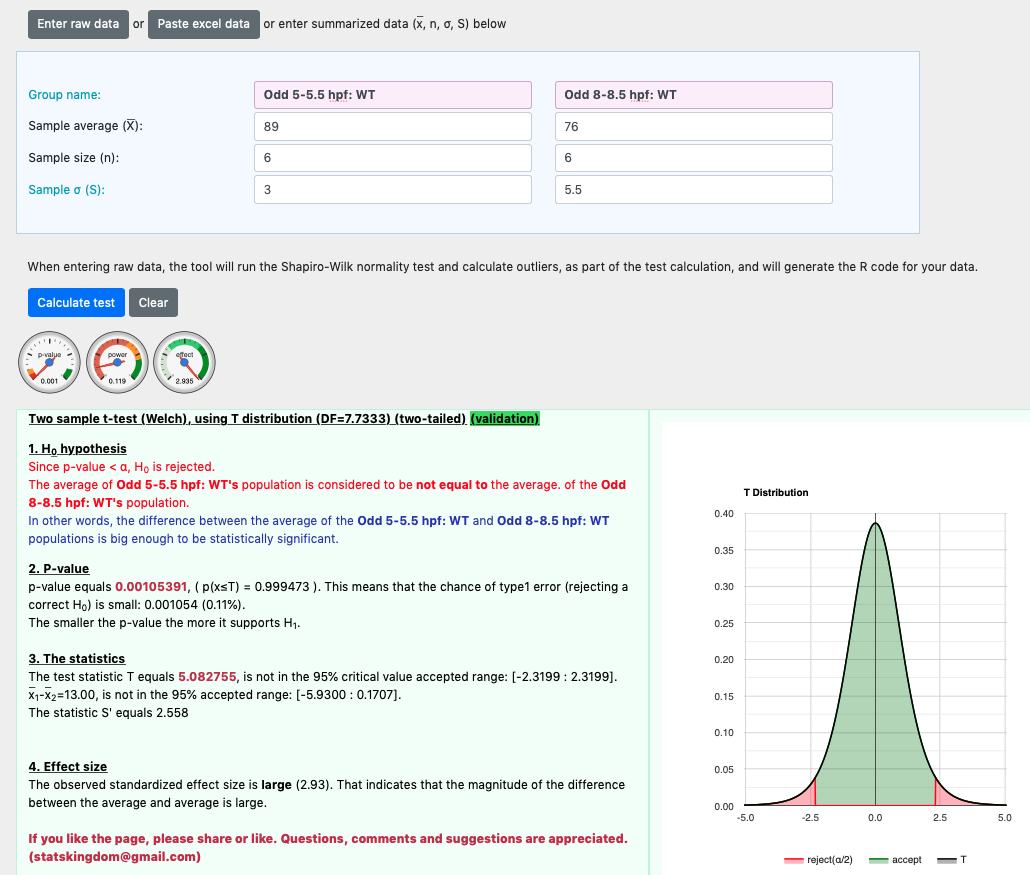
Odd in MP2 (5-5.5 hpf) versus dMP2 (8-8.5 hpf) in wild-type**

**Odd in MP2 (5-5.5 hpf) versus dMP2 (8-8.5 hpf) in *mid***


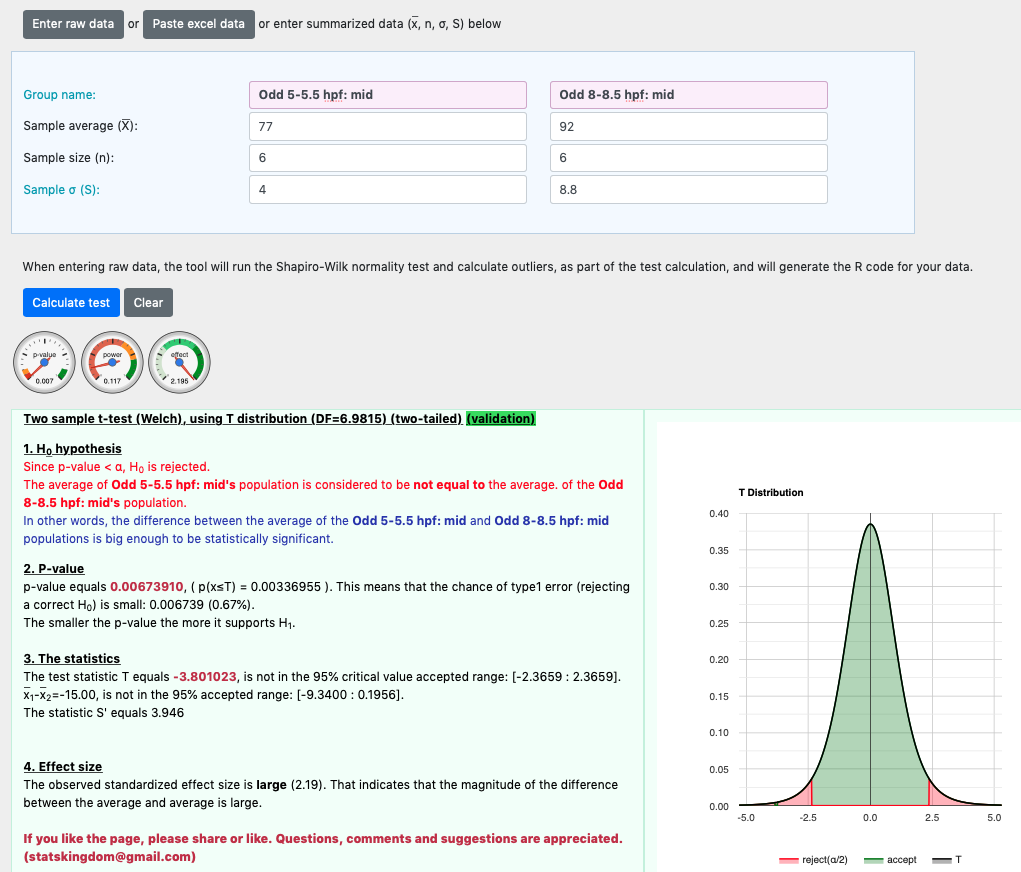

Supplement: S1 Statistics — Two Sample T-Test (Welch’s T-Test) analysis of the means between groups of datasets. Statistics: Wild-type control vs mid mutant: Cyclin E (5–5.5 hr) in MP2: wild-type versus mid: P = 0.631777 (H0 is accepted) Odd (5–5.5 hr) in MP2: wild-type versus mid: P = 0.0146960 (H0 is rejected) Cyclin E (5.5–6.0) in MP2: wild-type versus mid: P = 5.61231e-7(H0 is rejected) Odd (5.5–6.0) in MP2: wild-type versus mid: P = 0.285161 (H0 is accepted) Cyclin E (8–8.5) in dMP2: wild-type versus mid: P = 0.679337(H0 is accepted) Odd (8–8.5 hr) in dMP2: wild-type versus mid: P = 0. 00496460(H0 is rejected) Between 5.0–5.5 hpf vs 5.5–6.0, control and mid: Control—Cyclin E: P = 0.000155669 (H0 is rejected); Odd: P = 0.0865129 (Ho is accepted) mid—Cyclin E: P = 0.0000448175 (H0 is rejected); Odd: P = 0.00152362 (H0 is rejected) Between 5.5–6.0 hpf vs 8–8.5, control and mid: Control—Cyclin E: P = 0.432896 (H0 is accepted); Odd: P = 0.000408976 (Ho is rejected) mid—Cyclin E: P = 2.58473e-7 (H0 is rejected); Odd: P = 0.833928 (H0 is accepted) Between 5–5.5 hpf vs 8–8.5, control and mid: Control—Cyclin E: P = 0.0000181578 (H0 is accepted); Odd: P = 0.00105391(Ho is rejected) mid—Cyclin E: P = 0.0000790914 (H0 is rejected); Odd: P = 0.00673910(H0 is rejected). (DOCX) [file pgen.1009011.s006.docx]
